# Association between body mass index at different levels and risk of gastroesophageal reflux disease: a systematic review with dose-response meta-analysis

**DOI:** 10.3389/fphys.2025.1675457

**Published:** 2025-11-26

**Authors:** Mao Yiqing, Zhang Yangyang, Hu Lanshuo, Zhang Wenhao, An Yixin, Zhao Yingpan, Tang Xudong

**Affiliations:** 1 Graduate School, China Academy of Chinese Medical Sciences, Beijing, China; 2 Graduate School, Beijing University of Traditional Chinese Medicine, Beijing, China; 3 Institute of Digestive Diseases, Xiyuan Hospital of China Academy of Chinese Medical Sciences, Beijing, China

**Keywords:** body mass index, BMI, obesity, gastroesophageal reflux disease, prevalence, systematic review

## Abstract

**Objective:**

Although obesity is widely reported as an established risk factor for gastroesophageal reflux disease (GERD), divergent findings exist across studies. To address the problems of obsolete data and conflicting findings in previous studies, we conducted a systematic review and meta-analysis to explore the association between body mass index (BMI) and GERD.

**Methods:**

We searched Pubmed, Embase, Cochrane Library, and Web of Science for relevant studies, and obtained the prevalence of symptomatic gastroesophageal reflux (symptomatic GER) or GERD from the original studies for the different BMI groups. International BMI cut-off points were adopted to define underweight, overweight, and obesity. Meta-analysis of this association was performed by calculating the combined relative risk (RR) and 95% confidence intervals (95% CI) using a random-effects model. In addition, subgroup and dose-response analyses were performed to explore subgroup differences and the association between BMI and GERD.

**Results:**

Analysis of 43 papers (39 cross-sectional studies, 4 case-control studies) with a total of 484,219 study participants showed that BMI was associated with the risk of symptomatic GER (RR = 2.041, 95% CI: 1.507–2.763) and GERD (RR = 1.374, 95% CI: 1.260–1.499). The results of the meta-analysis across different BMI groups suggest that, overweight (BMI ≥25 kg/m^2^) was an important inflection point for the risk of the diseases. In subgroup analyses comparing obese and non-obese populations, we incorporated other obesity diagnostic indicators and found that these might be a significant source of heterogeneity (p = 0.015). Dose-response analysis showed that for every 10 kg/m^2^ increase in BMI, the risk of prevalence of gastroesophageal reflux disease increased by 68% (RR = 1.681, 95% CI: 1.326–2.131).

**Conclusion:**

Elevated BMI increases the risk of symptomatic GER and GERD, and BMI is positively and linearly correlated with the risk of GERD. Overweight is an important inflection point for disease risk. High-quality prospective cohort studies are needed to explore the causality between the two factors and underlying mechanisms in the future.

## 1 Preface

Gastroesophageal reflux disease (GERD) is a group of disorders in which gastric contents reflux into the esophagus, causing uncomfortable symptoms and/or complications (Vakil et al., 2007). A global population-based study (Eusebi et al., 2018) showed a prevalence of 13% for at least one episode of GERD symptoms per week; and an update of a systematic review of population-based studies on the epidemiology of GERD (El-Serag et al., 2014) showed that the global combined prevalence of GERD in all regions has increased since 1995 and that GERD is now more prevalent than ever before. The prevalence of GERD has increased in all regions of the world since 1995, especially in North America (18.1%–27.8%) and East Asia (2.5%–7.8%), and prevalence estimates show considerable geographic variation. The complex pathogenesis (Zheng et al., 2021) and clinical symptoms (Katz et al., 2022) of the disease not only inconvenience diagnosis and treatment in modern medicine, leading to a decline in quality of life, but also may lead to more serious clinical outcomes such as Barrett’s esophagus (BE) and esophageal cancer (Katzka et al., 2020). According to a U.S. digestive disease statistic, healthcare expenditures related to esophageal diseases accounted for about 10% of the total burden of digestive diseases in the United States in 2018 (Peery et al., 2022), which has become an important public health issue. Therefore, in order to develop individualized interventions and treatment plans that meet the specific needs of patients and optimize their clinical outcomes, it is important for clinicians to gain a deeper understanding of the association between relevant risk factors and GERD.

In recent years, as the incidence and prevalence of obesity have increased dramatically around the world, obesity has attracted attention as a risk factor for multiple systemic diseases such as hypertension, diabetes mellitus, and coronary artery disease (Chew et al., 2023). The latest report of the World Health Organization (WHO) points out that since 1990, the global prevalence of obesity has more than doubled. Body mass index (BMI) is the most widely circulated and accepted indicator of obesity, and WHO defines BMI≥25 kg/m^2^ as overweight, BMI≥30 kg/m^2^ as obese, of which BMI 30–34.9 kg/m^2^ as class I obese, BMI 35–39.9 kg/m^2^ as class II obese, BMI≥40 kg/m^2^ as class III obese.

Obesity is also considered an independent risk factor for GERD. From a pathophysiological point of view, changes in the anatomy of the stomach and esophagus that may be caused by obesity are closely related to the development of GERD: some studies have shown that anti-reflux barrier defects, such as lower esophageal sphincter (LES) dysfunction (Herbella and Patti, 2010), transient lower esophageal sphincter relaxation (TLESR) (Wu et al., 2007), and hiatal hernia (HH) (Che et al., 2013), as well as impaired esophageal clearance function (Valezi et al., 2018), including reduced salivary secretion (Cote-Daigneault et al., 2014), impaired esophageal motility (Koppman et al., 2007), along with transdiaphragmatic pressure gradient (TGP) (Del et al., 2021) and prolonged gastric emptying time (Quitadamo et al., 2018), are more common in obese patients. In addition, abnormal serum levels of cytokines such as leptin (Pardak et al., 2021) and adiponectin (El-Serag et al., 2006) in obese patients may also contribute to the increased risk of GERD. However, some studies have also reported contradictory views (Quiroga et al., 2006). From the therapeutic aspect, modern medicine suggests that lifestyle modifications such as weight loss urgently need more attention as a basic treatment for patients with GERD, however, the indication of obesity for weight loss interventions in terms of BMI ranges is not clear.

Although the relationship between obesity or BMI and the prevalence of GERD has received much attention, the vast majority of studies have focused on the prevalence of GERD after various types of bariatric surgery, with mixed conclusions regarding BMI and the risk of GERD. First, as the number of high-quality original studies related to GERD increases year by year, the existing relevant systematic evaluations and meta-analyses (Cai et al., 2012; Corley and Kubo, 2006) data are relatively old and lack further dose-response analyses, which makes it difficult to accurately quantify the influence of increasing BMI or obesity classification (e.g., overweight, class I obese, class II obese) on the prevalence risk of GERD, and can’t provide a basis for the development of individualized intervention thresholds. Secondly, previous studies have mostly ignored the heterogeneity of the population, such as the differences in body fat distribution among different races may lead to a greater sensitivity to GERD in the corresponding populations, but there is a lack of relevant subgroup analyses. In addition, early meta-analyses did not adequately control for confounders (e.g., diet, smoking, alcohol consumption, etc.), and the lack of an objective diagnostic basis for the included studies may have led to effect size bias. To provide a more focused review and discussion of the relationship between BMI and GERD, we conducted this study to measure the correlation between BMI and the risk of GERD prevalence by summarizing and pooling the available evidence from observational studies, to provide more specific lifestyle guidance for patients with GERD and those at risk of GERD.

## 2 Methods

### 2.1 Protocol and registration

This study was registered in the International Prospective Register of Systematic Reviews (PROSPERO: CRD42024563046). Also, this study followed the reporting guidelines (Page et al., 2021a) of Meta-Analysis of Observational Studies in Epidemiology (Stroup et al., 2000).

### 2.2 Literature search and inclusion criteria

The search strategy used in this study included four English databases, Pubmed, Embase, Cochrane Library, and Web of Science. The search was conducted without language restriction, and the search covered online articles from the creation of the databases to 16 September 2024. The search strategies and keywords used for each database are listed in Supplementary Tables S1–S4. We manually searched for all references cited in the selected literature and their associated systematic evaluations and also consulted with relevant experts to ensure that we did not omit any literature that matched the study topic, thus guaranteeing the comprehensiveness of the literature search.

Microsoft Excel (Microsoft Corporation, Redmond, WA, United States) and NoteExpress software (Beijing Aegean Lezhi Technology Co., Ltd., BJ, China) were used for the evaluation. Two recorders (MYQ and ZYY) independently assessed titles and abstracts to determine inclusion criteria, and full texts were reviewed in detail when abstracts were deemed potentially relevant. Any conflicts or disagreements between reviewers were considered and determined unanimously, with the involvement of a third recorder (HLS) where necessary. All three reviewers were professional researchers trained in systematic literature searches. We used the design principles of Patient, Ex-posure, Comparison, Outcome, and Study (PECOS) to determine the eligibility criteria for study inclusion (Page et al., 2021a; Page et al., 2021b), details of which can be found in Supplementary Table S5. The following inclusion criteria were applied:(a) Patient: patients with a diagnosis of GERD or symptomatic GER. Patients with symptomatic GER were included in the criteria to include as many relevant studies as possible; (b) Exposure: underweight, overweight, obese BMI; (c) Control: normal BMI; (d) Outcome: risk of prevalence of symptomatic GER and GERD; and (e) Study design: observational studies, such as retrospective or prospective cohort studies, case-control studies, and cross-sectional studies.

Exclusion criteria were as follows: (a) studies that did not include data on the association between obesity (or BMI) and GERD in the exposed group or the non-exposed group, or both; (b) duplicate publications or substudies of the included trials; (c) studies in which the full text was not available after contacting the authors; (d) studies with incomplete full-text data for which the odds ratio (OR), relative risk (RR), or hazard ratio (HR) could not be obtained; and (e) studies with a sample size of less than 10 in both the exposed and non-exposed groups. Studies were not restricted to study country and ethnicity.

### 2.3 Definitions

In the included studies, the diagnosis of GERD had to be made by one of the following routes: (a) Physician diagnosis: GERD was diagnosed by a qualified physician according to various guidelines or criteria based on clinical assessment and diagnostic methods; (b) Questionnaire-based diagnosis: the diagnosis of GERD was made through authoritative questionnaires, such as gastroesophageal reflux disease questionnaire (GerdQ) (Jones et al., 2009) and Frequency Scale for the Symptoms of GERD etc. ; (c) Symptoms that meet the Montreal definition and classification, in which GERD is defined as a condi-tion characterized by the presence of mild symptoms on 2 or more days per week or moderate to severe symptoms on more than 1 day per week. Moderate to severe symptoms are characteristic of the disease (Vakil et al., 2007); (d) 24-h esophageal pH/impedance monitoring: acid exposure time (AET) > 4.2% is used as a criterion for abnormal acid reflux, and AET is defined as the percentage of time that the esophageal pH is <4 in 24 h (Kahrilas and Quigley, 1996; Patel et al., 2015). This specific cut-off was selected in accordance with established consensus guidelines (Kahrilas and Quigley, 1996) from the time period of many of the included studies instead of the AET >6% standard from The 2018 Lyon Consensus for GERD diagnosis (Gyawali et al., 2018) to maximize consistency and data inclusion across our heterogeneous dataset, which spans several decades. Fourth, the diagnostic Symptomatic GER is defined as the presence of symptoms associated with the reflux of gastric contents into the esophagus, such as heartburn and reflux, but does not necessarily meet the specific diagnostic criteria for GERD.

In this meta-analysis, the classification of body weight status was primarily based on the international standard BMI cut-off points established by the WHO: underweight (<18.5 kg/m^2^), normal weight (18.5–24.9 kg/m^2^), overweight (25.0–29.9 kg/m^2^), and obese (≥30.0 kg/m^2^), Class Ⅰ Obese (BMI 30–34.9 kg/m^2^), and Class Ⅱ Obese and above (BMI≥ 35 kg/m^2^) (World Obesity Federation, 2025). This uniform application was a necessary methodological choice to ensure consistency and comparability across the diverse set of included studies from different geographical regions and time periods.

### 2.4 Data extraction

Relevant data were extracted from the selected studies using a structured table. Both transcribers (MYQ and ZYY) extracted data independently using a standardized form, and a third transcriber performed a rigorous quality check. We extracted the title, first author, year of publication, country/region, type of study, study interval, sample size, sex, age, participants, diagnostic method, number of cases, confounders, the BMI criteria for the categorization of weight and its RR, OR or HR and corresponding 95% CI. When a study reported both crude and adjusted forms, we extracted adjusted estimates. In addition, we selected estimates fully adjusted for confounders in studies reporting several multivariate models. In the overall analysis, for studies that provided several BMI values, we used participants’ baseline BMI.

### 2.5 Study quality and bias assessment

Two recorders (MYQ and ZYY) conducted the methodological quality assessment of the included studies 'back-to-back'. The Agency for Healthcare Research and Quality Scale (AHRQ) was used to score cross-sectional studies on a scale of 0–11. Scores ranged from 0–3, 4–7, and 8–11, indicating low, medium, and high quality, respectively (Viswanathan et al., 2008). The Newcastle-Ottawa Quality Assessment Scale (NOS) was used to score case-control studies on a scale of 0–9, and these scores were further categorized into three groups: 0–3, 4–6, and 7–9 corresponding to low, medium and high quality studies (Stang, 2010). Any disagreements between the two recorders (MYQ and ZYY) were resolved through discussion and consensus.

### 2.6 Data analysis

Meta-analysis of the study was performed using Stata 17 software (StataCorp LLC, TX, USA) as well as R (version 4.2.2; R Foundation for Statistical Computing, Vienna, Austria), and the significance threshold for all analyses was set at p < 0.05. In the overall analysis, data from all included studies were comprehensively pooled and analyzed to compare the risk of GERD and symptomatic GER between the highest and the lowest BMI groups using traditional meta-analysis methods, RR and their corresponding 95% CI were extracted for meta-analysis. The RR and 95% CI of the different BMI groups provided by the study were also analyzed separately for comparison. Other effect indicators, such as OR and HR, were transformed into RR using a validated formula (VanderWeele and Ding, 2017; Zhang and Yu, 1998). For cross-sectional studies, the Prevalence Ratio (PR) was treated as an approximation of the RR. This approach is methodologically justified for the study of GERD, because GERD is a chronic and highly prevalent condition with a relatively stable course over time. In such epidemiological contexts, the prevalence measured in a cross-sectional study closely reflects the cumulative incidence or long-term risk of the disease, thereby allowing the PR to serve as a valid estimate of the RR (Greenland, 1987). Dose-response meta-analysis was conducted when the number of studies with more than 3 intake categories was sufficient. The magnitude of heterogeneity was determined using the I^2^ statistic and Cochran’s Q value. In the presence of significant heterogeneity (I^2^ ≥ 50% or p < 0.05), a random effects model was used for pooled analyses of GERD and symptomatic GER prevalence; otherwise, fixed-effects models were employed.

A two-stage random-effects dose-response meta-analysis was performed to quantify the exposure-effect relationship between BMI and GERD/symptomatic GER. The restricted maximum likelihood (REML) method was applied to estimate the summary RRs and 95% CIs across contiguous exposure categories. To account for correlation within studies with multiple exposure groups, the covariance matrix was approximated using the Greenland and Longnecker’s method, which was a validated ap-proach for handling correlated risk estimates in aggregated data. We utilised restricted cubic splines with a three-knot model positioned at the 10th, 50th and 90th percentiles to construct a dose–response curve. Both linear and nonlinear models were evaluated, with the Akaike Information Criterion (AIC) applied to determine the optimal fit. The BMI was estimated as the mean of the grouped upper and lower dose limits. For open interval BMI, the same width was assumed between each group and this was used to estimate the open BMI interval. Both linear and non-linear analyses were employed.

Subgroup analyses and meta-regressions were performed for the following possible sources of heterogeneity depending on the study setting: type of study, year of publication, income level, country/region, clinical outcome, diagnostic method, quality of study, sample size, other obesity indicators, and confounders (sex, age, smoking, alcohol consumption, education level, dietary habits, medication history, physical activity). If the source of heterogeneity could not be determined, qualitative synthesis was performed using descriptive statistical methods. Sensitivity analyses were performed in traditional meta-analysis if possible. When the number of studies included in the outcome was ≥9, potential publication bias was detected using funnel plots, and asymmetry was tested with Egger’s test.

## 3 Result

### 3.1 Literature screening process

**FIGURE 1:**
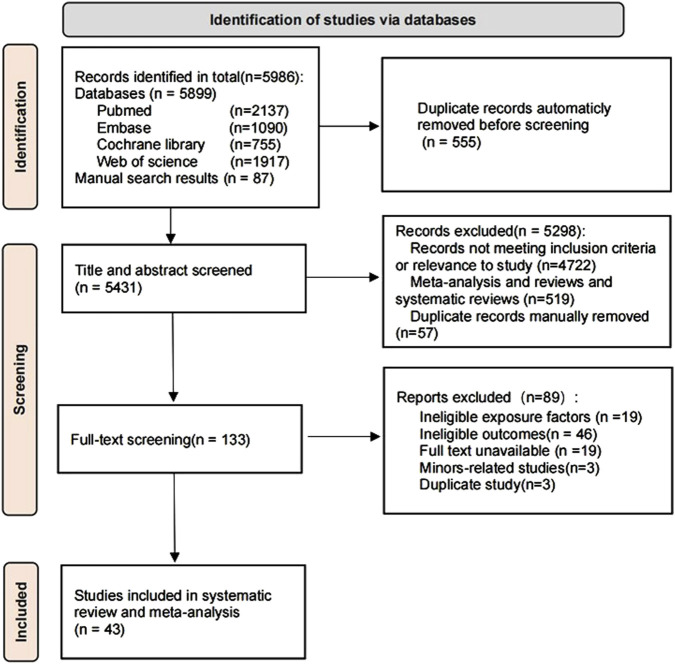
Literature flowchart and study selection, according to the PRISMA protocol.

The database search yielded a total of 5,899 literature records, 87 records were searched manually, and after using machine checking, 5,431 remained. After screening the titles and abstracts, 133 papers were downloaded, and after excluding papers due to unavailable outcome data (n = 46), exposure of non-interest (n = 19), unavailable full text (n = 19), involvement of minors (n = 3), and duplicated data (n = 3), finally, 43 studies (Chen et al., 2021; Rasool et al., 2021; Jacobson et al., 2006; Watanabe et al., 2003; Islami et al., 2014; Dore et al., 2008; Eslami et al., 2017; Baroni et al., 2023; Solhpour et al., 2008; Rosaida and Goh, 2004; de Oliveira et al., 2005; Chen et al., 2024; Tong et al., 2024; Lin et al., 2019; Yadegarfar et al., 2018; Ebrahimi-Mameghani et al., 2008; Cela et al., 2013; Nocon et al., 2006; Wenzl et al., 2021; Nilsson et al., 2003; Veugelers et al., 2006; El-Serag et al., 2005; Pandeya et al., 2012; Friedenberg et al., 2010; Otayf et al., 2022; Koul et al., 2018; Ghoshal et al., 2021; Sadeghi et al., 2024; Maleki et al., 2024; Chen et al., 2012; Wang et al., 2016; Hollenz et al., 2002; Bert et al., 2021; Odah et al., 2021; Hung et al., 2011; Ma et al., 2009; Liu et al., 2023; Sharma et al., 2011; Locke et al., 1999; Sadafi et al., 2024; Xue et al., 2021; Abed et al., 2024; Breckan et al., 2009) were considered eligible for data extraction and inclusion in this systematic review and meta-analysis, and the flowchart of the study screening process is shown in Figure 1. Of these, data from 28 articles (Chen et al., 2021; Rasool et al., 2021; Watanabe et al., 2003; Dore et al., 2008; Eslami et al., 2017; Baroni et al., 2023; Solhpour et al., 2008; Rosaida and Goh, 2004; Chen et al., 2024; Tong et al., 2024; Lin et al., 2019; Yadegarfar et al., 2018; Cela et al., 2013; Veugelers et al., 2006; Friedenberg et al., 2010; Otayf et al., 2022; Koul et al., 2018; Ghoshal et al., 2021; Sadeghi et al., 2024; Wang et al., 2016; Hollenz et al., 2002; Bert et al., 2021; Odah et al., 2021; Hung et al., 2011; Ma et al., 2009; Liu et al., 2023; Sadafi et al., 2024; Abed et al., 2024) met the definition of GERD in the current study.

### 3.2 Study characteristics

The characteristics of the included studies are summarized in Table 1. The included studies were published between 1999 and 2024. Four studies (Dore et al., 2008; Ebrahimi-Mameghani et al., 2008; Nilsson et al., 2003; Veugelers et al., 2006) adopted a case-control design, and the remaining studies (Chen et al., 2021; Rasool et al., 2021; Jacobson et al., 2006; Watanabe et al., 2003; Islami et al., 2014; Eslami et al., 2017; Baroni et al., 2023; Solhpour et al., 2008; Rosaida and Goh, 2004; Chen et al., 2024; Tong et al., 2024; Lin et al., 2019; Yadegarfar et al., 2018; Cela et al., 2013; Wenzl et al., 2021; Nilsson et al., 2003; El-Serag et al., 2005; Pandeya et al., 2012; Friedenberg et al., 2010; Otayf et al., 2022; Koul et al., 2018; Ghoshal et al., 2021; Sadeghi et al., 2024; Maleki et al., 2024; Chen et al., 2012; Wang et al., 2016; Hollenz et al., 2002; Bert et al., 2021; Odah et al., 2021; Ma et al., 2009; Liu et al., 2023; Sharma et al., 2011; Locke et al., 1999; Sadafi et al., 2024; Xue et al., 2021; Abed et al., 2024; Breckan et al., 2009) were cross-sectional. The sample sizes of individual studies ranged from 162 to 163,018 and included 79,755 patients (including 60,763 with GERD) and 404,464 asymptomatic participants. For GERS or GERD diagnosis, 4 studies (Eslami et al., 2017; Veugelers et al., 2006; Hollenz et al., 2002; Sadafi et al., 2024) relied on physician diagnosis, 3 studies (Baroni et al., 2023; Cela et al., 2013; Friedenberg et al., 2010) used the Montreal definition, 35 studies (Chen et al., 2021; Rasool et al., 2021; Jacobson et al., 2006; Watanabe et al., 2003; Islami et al., 2014; Dore et al., 2008; Solhpour et al., 2008; Rosaida and Goh, 2004; de Oliveira et al., 2005; Chen et al., 2024; Lin et al., 2019; Yadegarfar et al., 2018; Ebrahimi-Mameghani et al., 2008; Nocon et al., 2006; Wenzl et al., 2021; Nilsson et al., 2003; El-Serag et al., 2005; Pandeya et al., 2012; Otayf et al., 2022; Koul et al., 2018; Ghoshal et al., 2021; Sadeghi et al., 2024; Maleki et al., 2024; Chen et al., 2012; Wang et al., 2016; Bert et al., 2021; Odah et al., 2021; Hung et al., 2011; Ma et al., 2009; Liu et al., 2023; Sharma et al., 2011; Locke et al., 1999; Xue et al., 2021; Abed et al., 2024; Breckan et al., 2009) used authoritative questionnaires for diagnosis, and 1 study (Tong et al., 2024) used 24-h esophageal pH/impedance monitoring for diagnosis.

**TABLE 1:** Main characteristics of studies included in this meta-analysis.

Reference	Country	Design	Quality	Study interval	Sample size	Male/female (n)	Participant(n) (disease/ normal)	Diagnosis method	Obesity definition	Result
de Oliveira et al. (2005)	Brazil	Cross-sectional study	Median	1999–2000	3,934	1,691/2,243	1,232/2,702	Questionnaire diagnosis	—	GERS
Hollenz et al. (2002)	German	Cross-sectional study	Median	—	162	58/104	82/80	Physician’s diagnosis	BMI >25 kg/m^2^	GERD
Chen et al. (2021)	China	Cross-sectional study	Median	2018.7–2018.8	565	290/275	104/461	Questionnaire diagnosis	BMI ≥24 kg/m^2^	GERD
Rasool et al. (2021)	Pakistan	Cross-sectional study	Median	2019.11–2020.5	308	138/170	82/226	Questionnaire diagnosis	Normal: BMI 18.5–24.9 kg/m^2^ Overweight BMI 25–29.9 kg/m^2^ Obesity:BMI≥30 kg/m^2^	GERD
Jacobson et al. (2006)	America	Cross-sectional study	Median	2000	6,210	0/10,545	2,306/3,904	Questionnaire diagnosis	—	GERS
Watanabe et al. (2003)	Japan	Cross-sectional study	High	2001.7–2001.12	4,095	4,095/0	276/3,819	Questionnaire diagnosis	—	GERD
Dore et al. (2008)	Italy	Case-control study	High	—	500	169/331	300/200	Questionnaire diagnosis	BMI of ≥95th percentile for age and gender	GERD
Eslami et al. (2017)	Iran	Cross-sectional study	High	2014–2015	505	156/349	285/220	Physician’s diagnosis	BMI≥30 kg/m^2^	GERD
Baroni et al. (2023)	Italy	Cross-sectional study	High	2022.7.26–2023.5.16	1,146	83/1,063	106/1,040	Montreal definition	—	GERD
Solhpour et al. (2008)	Iran	Cross-sectional study	High	2006.5–2006.12	886	320/566	442/444	Questionnaire diagnosis	Overweight: BMI 25–30 kg/m^2^ Obese:BMI>30 kg/m^2^	GERD
Rosaida and Goh (2004)	Malaysia	Cross-sectional study	High	2001.6–2002.2	1,000	442/558	388/612	Questionnaire diagnosis	BMI>25 kg/m^2^	GERD
Chen et al. (2024)	China	Cross-sectional study	Median	—	121,583	43,698/77,885	16,664/104,919	Questionnaire diagnosis	—	GERD
Tong et al. (2024)	China	Cross-sectional study	Median	2015.1–2023.12	268	137/131	134/134	24h pH monitoring diagnosis	Overweight/Obese: BMI≥25 kg/m^2^	GERD
Lin et al. (2019)	China	Cross-sectional study	Median	2017.1–2017.12	3,352	1815/1,537	107/3,245	Questionnaire diagnosis	—	GERD
Ebrahimi-Mameghani et al. (2008)	Pakistan	Case-control study	High	2006.11–2007.3	217	57/160	106/111	Questionnaire diagnosis	Normal: BMI<25 kg/m^2^ Overweight: BMI25-29.9 kg/m^2^ Obesity:BMI≥30 kg/m^2^	GERS
Cela et al. (2013)	Albania	Cross-sectional study	Median	2012.3–2012.8	845	345/500	101/744	Montreal definition	—	GERD
Wenzl et al. (2021)	Austria	Cross-sectional study	Median	—	350	122/228	117/233	Questionnaire diagnosis	BMI≥25 kg/m^2^	GERS
Nilsson et al. (2003)	Norway	Case-control study	High	1984–1986 1995–1997	42,985	20,369/22,616	3,113/39,872	Questionnaire diagnosis	—	GERS
Veugelers et al. (2006)	Canada	Case-control study	High	2001.2–2003.2	244	143/101	142/102	Physician’s diagnosis	Underweight BMI <20 kg/m^2^ Normal weight:20≤BMI< 25 kg/m^2^ Overweight:25≤BM < 30 kg/m^2^ Obesity:BMI≥30 kg/m^2^	GERD
El-Serag et al. (2005)	America	Cross-sectional study	High	—	453	145/308	118/335	Questionnaire diagnosis	Normal: BMI<25 kg/m^2^ Overweight:BMI25-30 kg/m^2^ Obese BMI>30 kg/m^2^	GERS
Pandeya et al. (2012)	Austria	Cross-sectional study	High	2002–2005	902	593/309	175/727	Questionnaire diagnosis	—	GERS
Friedenberg et al. (2010)	America	Cross-sectional study	Median	—	503	195/308	129/374	Montreal definition	—	GERD
Ghoshal et al. (2021)	India	Cross-sectional study	Median	2011	2,774	1,573/1,201	298/2,476	Questionnaire diagnosis	BMI≥25 kg/m^2^	GERD
Sadeghi et al. (2024)	Iran	Cross-sectional study	High	2014–2023.3.3	163,018	72,943/90,075	34,434/128,584	Questionnaire diagnosis	—	GERD
Maleki et al. (2024)	Iran	Cross-sectional study	Median	—	10,255	4,149/6,106	2,826/7,429	Questionnaire diagnosis	—	GERS
Chen et al. (2012)	China	Cross-sectional study	High	2008.8–2009.8	8,051	3,097/5,734	150/7,901	Questionnaire diagnosis	—	GERS
Odah et al. (2021)	Saudi Arabia	Cross-sectional study	Median	2021.1	1,180	749/431	388/792	Questionnaire diagnosis	Underweight: BMI<18.5 kg/m^2^ Healthy weight:BMI18.5–24.9 kg/m^2^ Overweight:BMI 25–29.9 kg/m^2^ Obese BMI ≥30 kg/m^2^	GERD
Wang et al. (2016)	India	Cross-sectional study	Median	2010–2011	1,072	335/737	238/834	Questionnaire diagnosis	—	GERD
Hung et al. (2011)	China	Cross-sectional study	Median	2008.11–2009.10	1,238	565/673	310/928	Questionnaire diagnosis	—	GERD
Bert et al. (2021)	Italy	Cross-sectional study	Median	2019.6–2019.8	559	138/418	155/404	Questionnaire diagnosis	—	GERD
Ma et al. (2009)	China	Cross-sectional study	Median	—	919	410/509	57/862	Questionnaire diagnosis	—	GERD
Liu et al. (2023)	China	Cross-sectional study	Median	2012–2015	50,183	21,099/29,084	2,832/47,351	Questionnaire diagnosis	Normal and underweight:BMI<25 kg/m^2^ Overweight and obese BMI≥25 kg/m^2^	GERD
Sharma et al. (2011)	India	Cross-sectional study	High	2003.6–2005.1	4,039	2,902/1,137	653/3,386	Questionnaire diagnosis	—	GERS
Locke et al. (1999)	America	Cross-sectional study	High	1988–1991	956	457/499	304/652	Questionnaire diagnosis	—	GERS
Sadafi et al. (2024)	Iran	Cross-sectional study	Median	2023	9,631	4,625/5,006	1,058/8,573	Physician’s diagnosis	—	GERD
Xue et al. (2021)	China	Cross-sectional study	High	2014.1–2018.12	2027	1,016/1,011	556/1,471	Questionnaire diagnosis	—	GERS
Abed et al. (2024)	Palestinian	Cross-sectional study	Median	2023.11–2023.12	554	222/332	185/369	Questionnaire diagnosis	Underweight <18.5 kg/m^2^ Normal 18.5–24.9 kg/m^2^ Overweight 25–29.9 kg/m^2^ Obese ≥30 kg/m^2^	GERD
Breckan et al. (2009)	Norway	Cross-sectional study	Median	2004.12–2005.2	1,628	775/959	423/1,205	Questionnaire diagnosis	Underweight BMI<18.5 kg/m^2^ Pre-obese BMI 25–29.99 kg/m^2^ Obese class I BMI 30.00–34.99 kg/m^2^ Obese class II BMI 35.00–39.99 kg/m^2^ Obese class III BMI ≥40.00 kg/m^2^	GERS
Yadegarfar et al. (2018)	Iran	Cross-sectional study	Median	2014	1,106	522/575	717/389	Questionnaire diagnosis	—	GERD
Otayf et al. (2022)	Saudi Arabia	Cross-sectional study	Median	2021.3	953	384/569	220/733	Questionnaire diagnosis	—	GERD
Nocon et al. (2006)	German	Cross-sectional study	Median	—	5,240	2,448/2,792	1,250/3,990	Questionnaire diagnosis	Normal BMI≤24.9 kg/m^2^ Overweight BMI 25–29.9 kg/m^2^ Obese BMI>30 kg/m^2^	GERS
Koul et al. (2018)	Kashimir	Cross-sectional study	Median	2014.1–2015.1	2,600	—	529/2071	Questionnaire diagnosis	—	GERD
Islami et al. (2014)	Iran	Cross-sectional study	Median	2004.1–2008.6	25,223	11,295/13,928	5,663/19,560	Questionnaire diagnosis	—	GERS

— means unmentioned in text.

Abbreviations: GERS, symptomatic gastroesophageal reflux disease; GERD, gastroesophageal reflux disease; BMI, body mass index.

### 3.3 Risk of bias evaluation

The quality of studies included in the cross-sectional studies was evaluated using the AHRQ tool, 12 studies (Watanabe et al., 2003; Eslami et al., 2017; Baroni et al., 2023; Solhpour et al., 2008; Rosaida and Goh, 2004; El-Serag et al., 2005; Pandeya et al., 2012; Sadeghi et al., 2024; Chen et al., 2012; Sharma et al., 2011; Locke et al., 1999; Xue et al., 2021) were rated as high-quality and 27 (Chen et al., 2021; Rasool et al., 2021; Jacobson et al., 2006; Islami et al., 2014; de Oliveira et al., 2005; Chen et al., 2024; Tong et al., 2024; Lin et al., 2019; Yadegarfar et al., 2018; Cela et al., 2013; Nocon et al., 2006; Wenzl et al., 2021; Friedenberg et al., 2010; Otayf et al., 2022; Koul et al., 2018; Ghoshal et al., 2021; Maleki et al., 2024; Wang et al., 2016; Hollenz et al., 2002; Bert et al., 2021; Odah et al., 2021; Hung et al., 2011; Ma et al., 2009; Liu et al., 2023; Sadafi et al., 2024; Abed et al., 2024; Breckan et al., 2009) as moderate-quality. The quality of case-control studies was evaluated using the NOS tool, of which 4 studies (Dore et al., 2008; Ebrahimi-Mameghani et al., 2008; Nilsson et al., 2003; Veugelers et al., 2006) were all rated as high-quality, and the results of the quality evaluation analysis are shown in Supplementary Tables S6, S7. A summary of the quality of evidence obtained using the Grade of Recommendations Assessment, Development and Evaluation (GRADE) framework is provided in Supplementary Table S8.

## 4 Study outcomes

### 4.1 Main study outcome

**FIGURE 2:**
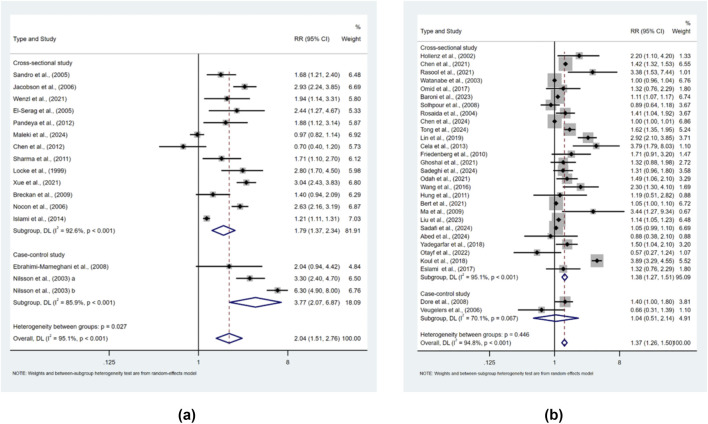
RRs for BMI of **(a)** symptomatic GER and **(b)** GERD.

When examining the association between obesity and the risk of symptomatic GER, random-effects models with 16 sets of data from a total of 15 studies showed high heterogeneity (I^2^ = 95.1%, p < 0.001), with a higher risk of disease in the higher BMI group (RR = 2.041, 95% CI 1.507–2.763); p < 0.001) (Figure 2a). When examining the association between obesity and the risk of GERD, a random-effects model with data from a total of 28 studies showed high heterogeneity (I^2^ = 94.80%, p < 0.001), with a higher risk of disease in the higher BMI group (RR = 1.374, 95% CI 1.260–1.499, p < 0.001) (Figure 2b).

#### 4.1.1 Subgroup analysis and meta-regression

To explore potential sources of heterogeneity in the relationship between BMI and GERD prevalence risk outcomes, we performed subgroup analyses in terms of study type, publication year, income level, country/region, study outcome, diagnostic method, quality of study, sample size, other obesity indicators, and confounders (sex, age, smoking, alcohol consumption, education level, dietary habits, medication history, and physical activity), and performed covariates of the above factors in a meta-regression.

According to the results of subgroup analysis, there were significant differences between subgroups according to year of publication (p = 0.001), country/region (p = 0.035), clinical outcome (p = 0.014), other obesity indicators (p < 0.001), and sample size (p = 0.038) (Table 2). Among the subgroups, the year of publication was before 2020 (RR = 1.888, 95%CI 1.506–2.366), the country/region was located in Europe (RR = 2.046, 95%CI 1.592–2.631) and North America (RR = 2.003, 95%CI 1.278–3.141), the clinical outcome was symptomatic GER (RR = 2.041, 95%CI 1.507–2.763), no other indicators of obesity were used (RR = 1.783, 95%CI 1.566–2.031), sample size >2000 (RR = 1.757, 95%CI 1.526–2.022), showed a stronger association compared to subgroups which publication year was in or after 2020 (RR = 1.257, 95%CI 1.151–1.373), publication region in Asia (RR = 1.425, 95% CI 1.284–1.581) and South America (RR = 1.680, 95% CI 1.193–2.366), study outcome of GERD (RR = 1.374, 95% CI 1.260–1.499), and the use of other obesity indicators (RR = 1.062, 95% CI 0.981–1.150), and sample size ≤2000 (RR = 1.452, 95% CI 1.300–1.623). In the subgroup analysis of confounders, there were statistically significant differences between subgroups for smoking (p = 0.035), education level (p = 0.003), dietary habits (p = 0.030), and medication history (p = 0.003), which may have a notable impact on the relationship between BMI and GERD prevalence, as shown in Table 2.

**TABLE 2:** Subgroup analyses of association between BMI and GERD according to study characteristics

	Subgroup	No. of study	Heterogeneity		p for between group	Result of subgroup analyses		Meta-regression
			I^2^	p		RR (95%CI)	P	
Study type					0.361			0.136
	Cross-sectional study	39	95.5%	<0.001		1.518 (1.397,1.650)	<0.001	
	Case-control study	5	94.9%	<0.001		2.165 (1.015,4.621)	0.046	
Publication year					**0.001**			**0.021**
	Before 2020	28	96.1%	<0.001		1.888 (1.506,2.366)	<0.001	
	In or after 2020	16	94.4%	<0.001		1.257 (1.151,1.373)	<0.001	
Income level					0.152			0.436
	Middle	26	96.0%	<0.001		1.526 (1.342,1.734)	<0.001	
	High	18	96.1%	<0.001		1.781 (1.505,2.108)	<0.001	
Country/region					**0.035**			0.103
	Asia	27	95.7%	<0.001		1.425 (1.284,1.581)	<0.001	
	Europe	11	97.0%	<0.001		2.046 (1.592,2.631)	<0.001	
	North America	5	73.3%	0.005		2.003 (1.278,3.141)	0.002	
	South America	1	—	—		1.680 (1.193,2.366)	0.003	
Study outcome					**0.014**			**0.028**
	GERD	28	95.0%	<0.001		1.374 (1.260,1.499)	<0.001	
	GERS	16	95.1%	<0.001		2.041 (1.507,2.763)	<0.001	
Diagnostic method					0.287			0.726
	Questionnaire diagnosis	36	96.7%	<0.001		1.677 (1.506,1.869)	<0.001	
	Physician’s diagnosis	4	55.8%	0.079		1.170 (0.824,1.661)	0.380	
	Montreal definition	3	83.4%	0.002		1.787 (0.891,3.581)	0.102	
	24-h pH monitoring diagnosis	1	—	—		1.621 (1.350,1.946)	<0.001	
Other obesity indicators					**<0.001**			**0.016**
	Yes	7	78.8%	<0.001		1.062 (0.981,1.150)	0.135	
	No	37	95.7%	<0.001		1.783 (1.566,2.031)	<0.001	
Study quality					0.682			0.929
	Median	27	96.2%	<0.001		1.586 (1.414,1.780)	<0.001	
	High	17	95.8%	<0.001		1.664 (1.365,2.029)	<0.001	
Sample size					**0.038**			0.398
	≤2000	26	82.0%	<0.001		1.452 (1.300,1.623)	<0.001	
	>2000	18	98.1%	<0.001		1.757 (1.526,2.022)	<0.001	
Confounders								
Sex					0.460			0.605
	Yes	27	94.8%	<0.001		1.489 (1.360,1.630)	<0.001	
	No	9	97.5%	<0.001		2.077 (1.140,3.785)	0.017	
	None	8	95.2%	<0.001		1.262 (0.744,2.141)	0.388	
Age					0.300			0.342
	Yes	30	96.2%	<0.001		1.586 (1.450,1.734)	<0.001	
	No	6	62.8%	0.02		2.030 (1.403,2.939)	<0.001	
	None	8	95.7%	<0.001		1.262 (0.744,2.141)	0.388	
Smoking					**0.035**			0.103
	Yes	25	96.8%	<0.001		1.747 (1.556,1.962)	<0.001	
	No	11	82.7%	<0.001		1.394 (1.214,1.601)	<0.001	
	None	8	95.7%	<0.001		1.262 (0.744,2.141)	0.388	
Alcohol consumption					0.214			0.532
	Yes	16	96.4%	<0.001		1.527 (1.359,1.717)	<0.001	
	No	20	94.5%	<0.001		1.798 (1.509,2.142)	<0.001	
	None	8	95.7%	<0.001		1.262 (0.744,2.141)	0.388	
Education level					**0.003**			0.523
	Yes	11	75.4%	<0.001		1.319 (1.167,1.490)	<0.001	
	No	25	96.8%	<0.001		1.745 (1.559,1.953)	<0.001	
	None	8	95.7%	<0.001		1.262 (0.744,2.141)	0.388	
Dietary habits					**0.030**			**0.042**
	Yes	10	95.2%	<0.001		2.117 (1.648,2.719)	<0.001	
	No	26	94.9%	<0.001		1.497 (1.361,1.647)	<0.001	
	None	8	95.7%	<0.001		1.262 (0.744,2.141)	0.388	
Medication history					**0.003**			**0.005**
	Yes	6	87.2%	<0.001		3.005 (1.957,4.613)	<0.001	
	No	30	94.2%	<0.001		1.417 (1.310,1.532)	<0.001	
	None	8	95.7%	<0.001		1.262 (0.744,2.141)	0.388	
Physical activity					0.481			0.156
	Yes	7	96.6%	<0.001		1.973 (1.197,3.251)	0.008	
	No	29	94.5%	<0.001		1.633 (1.463,1.823)	<0.001	
	None	8	95.7%	<0.001		1.262 (0.744,2.141)	0.388	

Abbreviations: 95%CI, 95% confidence interval; GERS, symptomatic gastroesophageal reflux disease; GERD, gastroesophageal reflux disease; RR, relative risk. The bolded entries in the table indicate p < 0.05, suggesting statistically significant differences.

Meta-regression results indicated that the year of publication, clinical outcome, other obesity indicators, dietary habits and medication history among confounders may influence the magnitude of heterogeneity, as shown in Table 2.

#### 4.1.2 Publication bias

**FIGURE 3:**
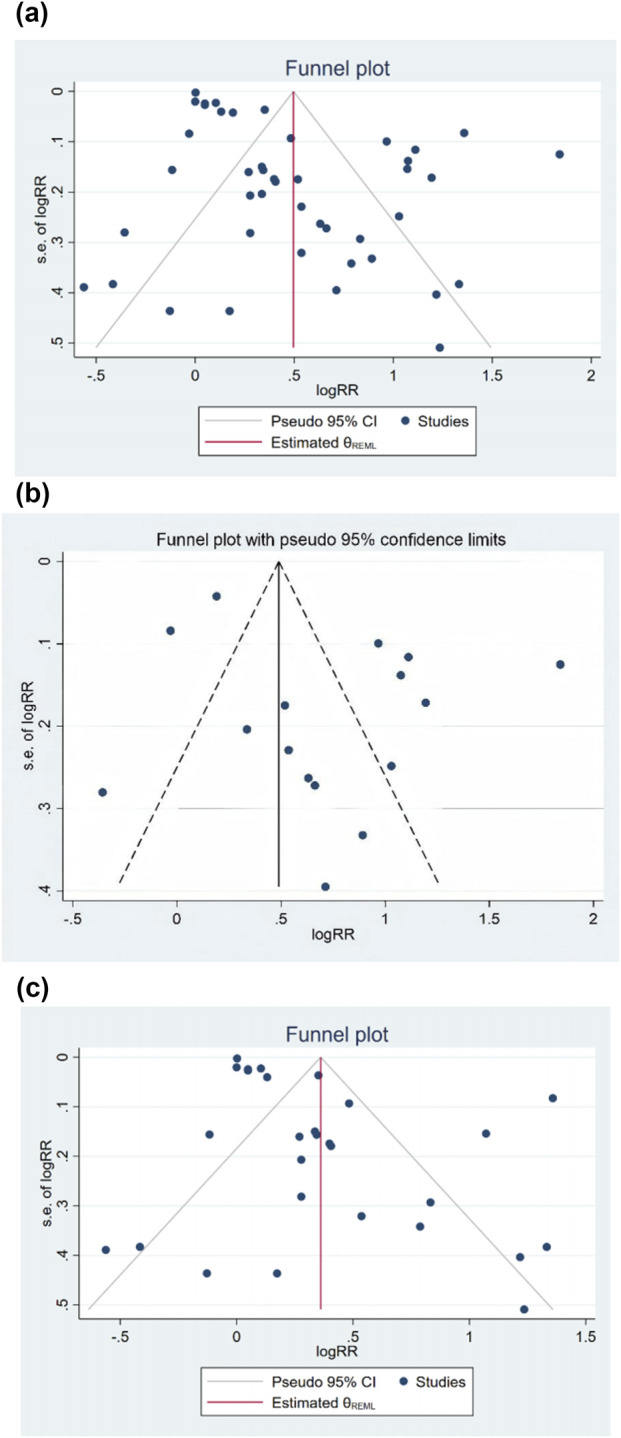
**(a)** The risk of symptomatic gastroesophageal reflux disease and gastroesophageal reflux disease associated with BMI was included in the study funnel plot. **(b)** The risk of symptomatic gastroesophageal reflux disease associated with BMI was in-cluded in the study funnel plot. **(c)** The risk of gastroesophageal reflux disease associated with BMI was included in the study funnel plot.

In BMI and symptomatic GER and GERD prevalence risk outcomes, the number of included studies was 43 (including 44 sets of study data), we drew a funnel plot to detect publication bias (Figure 3a) and found that the two sides of the funnel plot were asymmetric, which was confirmed to have a certain publication bias by Egger’s test (p < 0.001).

In the outcome of BMI and risk of developing symptomatic GER, the number of included studies was 15 (containing data from 16 study groups), and publication bias was detected by plotting a funnel plot (Figure 3b), which was found to be essentially symmetrical on both sides of the funnel plot, and Egger’s test confirmed that there was no publication bias (p = 0.096).

In the outcome of BMI and risk of developing GERD, the number of included studies was 28, and publication bias was detected by plotting the funnel plot (Figure 3c), which was asymmetric on both sides, and the Egger’s test confirmed that there was some publication bias (p = 0.001).

#### 4.1.3 Sensitivity analysis

In BMI and symptomatic GER and GERD prevalence risk outcomes, Egger’s test suggested the presence of publication bias (p < 0.001), but the trim-and-fill method of analysis did not identify missing studies to be filled in (Imputed = 0), and the corrected effect size was statistically significant and remained stable (Supplementary Figure S4). In the outcome of BMI and risk of developing GERD, although Egger’s test suggested the presence of publication bias (p = 0.001), the outcome of trim-and-fill method of analysis is similar (Imputed = 0), and the corrected effect size was still statistically significant and remained stable (Supplementary Figure S5). The direction and strength of the combined effect sizes were consistent across all analyses, supporting the reliability of the BMI- symptomatic GER and GERD association. In the future, prospective registry studies are needed to reduce publication bias and use multi-method cross-validation to enhance the robustness of the findings. Although trim-and-fill method suggested robust results, the significance of Egger’s test cautioned us to interpret findings with caution, as publication bias or other small-sample effects cannot be entirely ruled out.

### 4.2 Results of other studies

The risk of symptomatic GER and GERD prevalence in people with different BMI classifications were compared separately, and the results were as follows in Table 3.

**TABLE 3:** Summary of meta-analysis on the association between BMI and prevalence of symptomatic GER or GERD.

	Comparator	Studies	Relative risk (95% CI)	Heterogeneity	
				I^2^	p-value
Underweight	Normal BMI	6	0.90 (0.71,1.15)	31.7%	0.198
Subgroup Analysis					
Study type	Cross-sectional study	5	0.91 (0.70,1.19)	42.9%	0.136
	Case-control study	1	0.53 (0.08,3.59)	0.0%	<0.001
Publication year	Before 2020	4	0.84 (0.66,1.07)	29.0%	0.238
	In or after 2020	2	1.28 (0.75,2.18)	12.4%	0.285
Income level	Middle	3	0.95 (0.83,1.10)	0.0%	0.899
	High	3	0.91 (0.46,1.83)	67.1%	0.048
Study outcome	GERD	4	1.16 (0.74,1.83)	0.0%	0.489
	GERS	2	0.83 (0.59,1.17)	73.7%	0.051
Quality of study	Median	5	0.91 (0.70,1.19)	42.9%	0.136
	High	1	0.53 (0.08,3.59)	0.0%	0.582
Sample size	≤2000	4	1.16 (0.74,1.83)	0.0%	0.489
	>2000	2	0.83 (0.59,1.17)	73.7%	0.051
Normal BMI	Underweight	5	1.14 (0.91,1.43)	41.5%	0.145
Subgroup Analysis					
Study type	Cross-sectional study	5	1.14 (0.91,1.43)	41.5%	0.145
	Case-control study	0	—	—	—
Publication year	Before 2020	4	1.25 (1.05,1.49)	0.0%	0.613
	In or after 2020	1	0.71 (0.45,1.13)	0.0%	<0.001
Income level	Middle	4	1.25 (1.05,1.49)	0.0%	0.613
	High	1	0.71 (0.45,1.13)	0.0%	<0.001
Study outcome	GERD	3	1.04 (0.63,1.74)	65.5%	0.055
	GERS	3	1.21 (0.98,1.48)	0.0%	0.381
Quality of study	Median	4	1.07 (0.79,1.45)	48.2%	0.122
	High	1	1.31 (0.99,1.73)	0.0%	<0.001
Sample size	≤2000	2	0.78 (0.52,1.17)	0.0%	0.433
	>2000	3	1.26 (1.06,1.51)	0.0%	0.447
non-underweight	Underweight	1	1.20 (0.95,1.50)	—	—
Overweight	Non-overweight	21	1.49 (1.29,1.73)	88.40%	**<0.001**
Subgroup Analysis					
Study type	Cross-sectional study	18	1.38 (1.21,1.57)	81.2%	<0.001
	Case-control study	3	2.14 (1.93,2.37)	0.0%	0.465
Publication year	Before 2020	15	1.59 (1.30,1.94)	81.0%	<0.001
	In or after 2020	6	1.30 (1.10,1.54)	88.3%	<0.001
Income level	Middle	12	1.37 (1.17,1.60)	87.0%	<0.001
	High	9	1.72 (1.42,2.08)	61.8%	0.007
Study outcome	GERD	11	1.42 (1.21,1.67)	83.7%	<0.001
	GERS	10	1.60 (1.20,2.13)	91.1%	<0.001
Quality of study	Median	11	1.45 (1.22,1.71)	86.9%	<0.001
	High	10	1.53 (1.21,1.95)	83.6%	<0.001
Sample size	≤2000	14	1.44 (1.27,1.65)	50.8%	0.015
	>2000	7	1.52 (1.12,2.06)	95.8%	<0.001
Confounders					
Sex	Yes	14	1.43 (1.24,1.65)	80.2%	<0.001
	No	5	1.97 (1.57,2.47)	62.5%	0.031
	None	2	1.06 (0.81,1.40)	50.4%	0.156
Age	Yes	16	1.44 (1.24,1.67)	86.6%	<0.001
	No	3	2.80 (2.17,3.60)	0.0%	0.748
	None	2	1.06 (0.81,1.40)	50.4%	0.156
Smoking	Yes	13	1.51 (1.27,1.81)	90.6%	<0.001
	No	6	1.66 (1.28,2.14)	45.8%	0.1
	None	2	1.06 (0.81,1.40)	50.4%	0.156
Alcohol consumption	Yes	5	1.34 (1.18,1.53)	23.1%	0.267
	No	14	1.67 (1.34,2.07)	90.2%	<0.001
	None	2	1.06 (0.81,1.40)	50.4%	0.156
Overweight	Underweight	5	1.44 (1.04,1.98)	67.2%	**0.016**
Subgroup Analysis					
Study type	Cross-sectional study	5	1.44 (1.04,1.98)	67.2%	0.016
	Case-control study	0	—	—	—
Publication year	Before 2020	4	1.59 (1.17,2.17)	62.3%	0.047
	In or after 2020	1	0.89 (0.50,1.59)	0.0%	<0.001
Income level	Middle	4	1.59 (1.17,2.17)	62.3%	0.047
	High	1	0.89 (0.50,1.59)	0.0%	<0.001
Study outcome	GERD	3	1.19 (0.60,2.39)	79.0%	0.008
	GERS	2	1.59 (1.11,2.29)	62.2%	0.104
Quality of study	Median	4	1.28 (0.84,1.95)	70.9%	0.016
	High	1	1.91 (1.40,2.60)	0.0%	<0.001
Sample size	≤2000	2	0.85 (0.53,1.36)	0.0%	0.762
	>2000	3	1.74 (1.33,2.27)	54.1%	0.113
Overweight	Normal BMI	10	1.51 (1.21,1.89)	88.0%	**<0.001**
Subgroup Analysis					
Study type	Cross-sectional study	9	1.59 (1.27,1.99)	88.6%	<0.001
	Case-control study	1	0.66 (0.33,1.33)	0.0%	<0.001
Publication year	Before 2020	8	1.48 (1.15,1.91)	90.6%	<0.001
	In or after 2020	2	1.59 (1.19,2.11)	0.0%	0.741
Income level	Middle	4	1.21 (1.12,1.31)	0.0%	0.508
	High	6	1.77 (1.43,2.18)	75.5%	0.001
Study outcome	GERD	5	1.20 (0.91,1.60)	45.8%	0.117
	GERS	5	1.77 (1.30,2.41)	93.8%	<0.001
Quality of study	Median	9	1.59 (1.27,1.99)	88.6%	<0.001
	High	1	0.66 (0.33,1.33)	0.0%	<0.001
Sample size	≤2000	6	1.29 (1.03,1.61)	41.2%	0.131
	>2000	4	1.84 (1.28,2.65)	95.4%	<0.001
Confounders					
Sex	Yes	5	1.42 (1.10,1.83)	80.5%	<0.001
	No	3	1.88 (1.29,2.74)	87.7%	<0.001
	None	2	1.07 (0.47,2.46)	78.9%	0.030
Age	Yes	6	1.65 (1.23,2.21)	92.6%	<0.001
	No	2	1.28 (0.97,1.68)	2.5%	0.311
	None	2	1.07 (0.47,2.46)	78.9%	0.030
Smoking	Yes	7	1.66 (1.26,2.18)	91.2%	<0.001
	No	1	1.20 (0.90,1.60)	0.0%	<0.001
	None	2	1.07 (0.47,2.46)	78.9%	0.030
Alcohol consumption	Yes	5	1.67 (1.19,2.35)	94.1%	<0.001
	No	3	1.38 (1.14,1.67)	0.0%	0.409
	None	2	1.07 (0.47,2.46)	78.9%	0.030
Obese	Underweight	5	1.60 (0.85,3.02)	91.90%	**<0.001**
Subgroup Analysis					
Study type	Cross-sectional study	5	1.60 (0.85,3.02)	91.9%	<0.001
	Case-control study	0	—	—	—
Publication year	Before 2020	4	2.02 (1.11,3.67)	90.4%	<0.001
	In or after 2020	1	0.57 (0.27,1.22)	0.0%	<0.001
Income level	Middle	4	2.02 (1.11,3.67)	90.4%	<0.001
	High	1	0.57 (0.27,1.22)	0.0%	<0.001
Study outcome	GERD	3	1.44 (0.39,5.33)	93.2%	<0.001
	GERS	2	1.69 (1.29,2.22)	0.0%	0.951
Quality of study	Median	4	1.55 (0.71,3.40)	93.1%	<0.001
	High	1	1.71 (1.09,2.68)	0.0%	<0.001
Sample size	≤2000	2	0.80 (0.39,1.65)	37.0%	0.208
	>2000	3	2.28 (1.20,4.35)	92.5%	<0.001
Obese	Normal BMI	7	1.76 (1.24,2.49)	78.00%	**<0.001**
Subgroup Analysis					
Study type	Cross-sectional study	6	1.95 (1.42,2.69)	73.3%	0.002
	Case-control study	1	0.66 (0.31,1.40)	0.0%	<0.001
Publication year	Before 2020	5	1.66 (1.05,2.61)	82.3%	<0.001
	In or after 2020	2	2.07 (0.94,4.55)	71.2%	0.062
Income level	Middle	3	2.31 (1.23,4.36)	60.6%	0.079
	High	4	1.50 (0.92,2.45)	86.3%	<0.001
Study outcome	GERD	5	1.64 (1.07,2.49)	64.4%	0.024
	GERS	2	1.97 (1.06,3.64)	87.1%	0.005
Quality of study	Median	6	1.95 (1.42,2.69)	73.3%	0.002
	High	1	0.66 (0.31,1.40)	0.0%	<0.001
Sample size	≤2000	6	1.56 (1.13,2.15)	56.2%	0.044
	>2000	1	2.63 (2.16,3.20)	0.0%	<0.001
Obese	Non-overweight	8	1.61 (1.09,2.40)	84.50%	**<0.001**
Subgroup Analysis					
Study type	Cross-sectional study	7	1.57 (1.03,2.40)	86.1%	<0.001
	Case-control study	1	2.04 (0.94,4.42)	0.0%	<0.001
Publication year	Before 2020	7	1.80 (1.09,2.97)	83.1%	<0.001
	In or after 2020	1	0.97 (0.82,1.14)	0.0%	<0.001
Income level	Middle	6	1.35 (0.91,1.99)	80.3%	<0.001
	High	2	2.67 (1.80,3.94)	0.0%	0.740
Study outcome	GERD	3	1.89 (0.77,4.64)	88.5%	<0.001
	GERS	5	1.52 (0.88,2.61)	85.3%	<0.001
Quality of study	Median	3	1.93 (0.81,4.56)	89.5%	<0.001
	High	5	1.50 (0.84,5.68)	84.1%	<0.001
Sample size	≤2000	6	2.11 (1.24,3.58)	82.0%	<0.001
	>2000	2	0.92 (0.73,1.17)	19.5%	0.265
Obese	Non-obese	9	1.32 (1.04,1.68)	90.3%	**<0.001**
Subgroup Analysis					
Study type	Cross-sectional study	8	1.31 (1.01,1.71)	91.4%	<0.001
	Case-control study	1	1.40 (1.04,1.88)	0.0%	<0.001
Publication year	Before 2020	6	1.18 (0.98,1.43)	49.7%	0.077
	In or after 2020	3	1.53 (0.90,2.60)	97.1%	<0.001
Income level	Middle	8	1.31 (1.01,1.71)	91.4%	<0.001
	High	1	1.40 (1.04,1.88)	0.0%	<0.001
Study outcome	GERD	3	1.49 (1.19,1.87)	0.0%	0.539
	GERS	6	1.25 (0.93,1.68)	93.5%	<0.001
Quality of study	Median	3	1.07 (0.99,1.16)	6.8%	0.342
	High	6	1.49 (0.98,2.29)	88.7%	<0.001
Sample size	≤2000	3	1.49 (1.19,1.87)	0.0%	0.509
	>2000	6	1.25 (0.93,1.68)	93.5%	<0.001
Other obesity indicators	Yes	7	1.12 (1.00,1.26)	47.8%	0.074
	No	2	2.08 (0.92,4.70)	86.7%	0.006
Confounders					
Sex	Yes	5	1.35 (0.85,2.16)	94.2%	<0.001
	No	0	—	—	—
	None	4	1.18 (0.99,1.42)	59.7%	0.059
Age	Yes	5	1.35 (0.85,2.16)	94.2%	<0.001
	No	0	—	—	—
	None	4	1.18 (0.99,1.42)	59.7%	0.059
Smoking	Yes	4	1.34 (0.74,2.42)	95.6%	<0.001
	No	1	1.40 (1.04,1.88)	0.0%	<0.001
	None	4	1.18 (0.99,1.42)	59.7%	0.059
Alcohol consumption	Yes	4	1.34 (0.74,2.42)	95.6%	<0.001
	No	1	1.40 (1.04,1.88)	0.0%	<0.001
	None	4	1.18 (0.99,1.42)	59.7%	0.059
Class Ⅰ Obese	Normal BMI	5	2.66 (2.04,3.48)	82.4%	**<0.001**
Subgroup Analysis					
Study type	Cross-sectional study	3	2.02 (1.25,3.26)	78.7%	0.009
	Case-control study	2	3.47 (2.77,4.34)	71.5%	0.064
Publication year	Before 2020	5	2.66 (2.04,3.48)	82.4%	<0.001
	In or after 2020	0	—	—	—
Income level	Middle	0	—	—	—
	High	5	2.66 (2.04,3.48)	82.4%	<0.001
Study outcome	GERD	1	1.63 (0.93,2.86)	0.0%	<0.001
	GERS	4	2.87 (2.21,3.74)	83.0%	<0.001
Quality of study	Median	2	2.32 (1.33,4.05)	72.1%	0.058
	High	3	2.80 (1.93,4.06)	88.2%	<0.001
Sample size	≤2000	2	1.57 (1.13,2.19)	0.0%	0.873
	>2000	3	3.30 (2.78,3.91)	61.7%	0.073
Class Ⅰ and Ⅱ obese	Underweight	1	1.22 (0.95,1.56)		
Class Ⅱ obese and above	Non-overweight	4	2.98 (1.60,5.53)	89.9%	**<0.001**
Subgroup Analysis					
Study type	Cross-sectional study	2	1.81 (1.21,2.70)	0.0%	0.819
	Case-control study	2	4.61 (2.45,8.68)	89.2%	0.002
Publication year	Before 2020	4	2.98 (1.60,5.53)	89.9%	<0.001
	In or after 2020	0	—	—	—
Income level	Middle	0	—	—	—
	High	4	2.98 (1.60,5.53)	89.9%	<0.001
Study outcome	GERD	1	1.71 (0.91,3.21)	0.0%	<0.001
	GERS	3	3.50 (1.81,6.78)	90.7%	<0.001
Quality of study	Median	1	1.71 (0.91,3.21)	0.0%	<0.001
	High	3	3.50 (1.81,6.78)	90.7%	<0.001
Sample size	≤2000	2	1.81 (1.21,2.70)	0.0%	0.819
	>2000	2	4.61 (2.45,8.68)	89.2%	0.002
Class Ⅱ obese and above	Normal BMI	1	2.93 (2.24,3.85)		
Class Ⅲ obese and above	Underweight	1	1.31 (0.96,1.80)		

— means unmentioned in text.

Abbreviations: GERS, symptomatic gastroesophageal reflux disease; GERD, gastroesophageal reflux disease; BMI, body mass index; 95%CI, 95% confidence interval. The bolded entries in the table indicate p < 0.05, suggesting statistically significant differences.

#### 4.2.1 Underweight (BMI<18.5 kg/m^2^)

**FIGURE 4:**
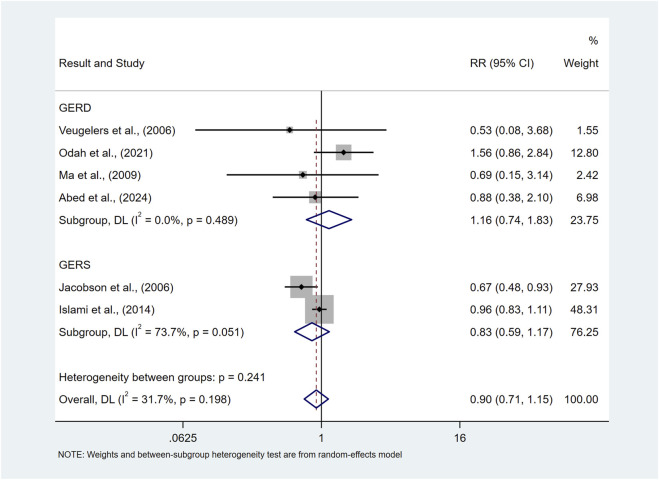
Forest plot to examine effect size and data dispersion in the publications in underweight and normal BMI groups.

Our findings showed that there was no significant difference in the risk of prevalence of symptomatic GER and GERD in the underweight population compared to normal BMI (RR = 0.90, 95%CI 0.71–1.15; I^2^ 31.7%) as shown in Figure 4.

#### 4.2.2 Normal BMI (BMI 18.5–24.9 kg/m^2^)

**FIGURE 5:**
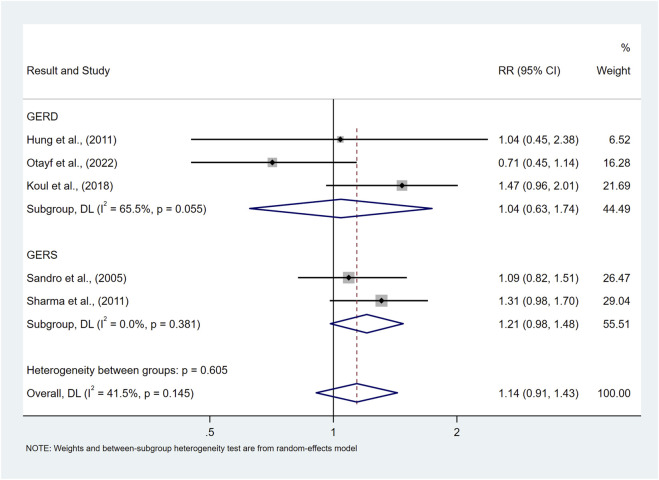
Forest plot to examine effect size and data dispersion in the publications in normal BMI and underweight groups.

Our findings showed that there was no significant difference in the risk of prevalence of symptomatic GER and GERD in the normal BMI population compared to the underweight population (RR = 1.14, 95%CI 0.91–1.43; I^2^ 41.5%) as shown in Figure 5.

#### 4.2.3 Overweight (BMI 25–29.9 kg/m^2^)

**FIGURE 6:**
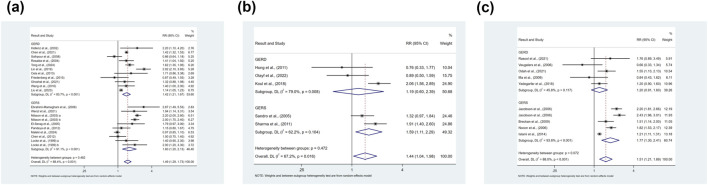
**(a)** Forest plot to examine effect size and data dispersion in the publications in overweight and non-overweight groups. **(b)** Forest plot to examine effect size and data dispersion in the publications in overweight and underweight groups. **(c)** Forest plot to examine effect size and data dispersion in the publications in overweight and normal BMI groups.

The overweight category was analyzed in 3 ways, 1) overweight vs. non-overweight population, 2) overweight vs. underweight population, and 3) over-weight vs. normal weight population. Our findings showed that being overweight was associated with an elevated risk of developing symptomatic GER and GERD compared with the non-overweight population (RR = 1.49, 95% CI 1.29–1.73; I^2^ 88.4%), as shown in Figure 6a. There was significant heterogeneity among studies, and to explore the sources of heterogeneity, the study was analyzed in terms of the type of study, year of publication, level of income, clinical outcomes, study quality, sample size and confounders (sex, age, smoking, alcohol consumption) were analyzed in subgroups, and it was found that the type of study might be the source of heterogeneity. Meta-regression with the above factors as covariates led to the same conclusion that study type might be a factor influencing the magnitude of heterogeneity (p = 0.022). For publication bias, the inverted funnel plot was symmetric (Supplementary Figure S1), which can be demonstrated by a nonsignificant Egger’s test (p = 0.358).

Being overweight was associated with an elevated risk of prevalence of symptomatic GER and GERD compared with underweight populations (RR = 1.44, 95% CI 1.04–1.98; I^2^ 67.2%), as shown in Figure 6b. The results of the subgroup analyses suggest that the variable of sample size may be a central source of heterogeneity, but meta-regression did not identify a source of heterogeneity. Due to the limited number of studies, subgroup analyses as well as publication bias analyses were not performed for confounders.

Being overweight was associated with an elevated risk of prevalence of symptomatic GER and GERD compared with the normal BMI population (RR = 1.51, 95%CI 1.21–1.89; I^2^ 88.0%), as shown in Figure 6c. The results of subgroup analyses suggested that the type of study, level of income, and study quality might be a source of heterogeneity, but meta-regression did not find a source of heterogeneity. For publication bias, the inverted funnel plot was symmetric (Supplementary Figure S2), which could be demonstrated by a non-significant Egger’s test (p = 0.554).

#### 4.2.4 Obesity (BMI ≥30 kg/m^2^)

**FIGURE 7:**
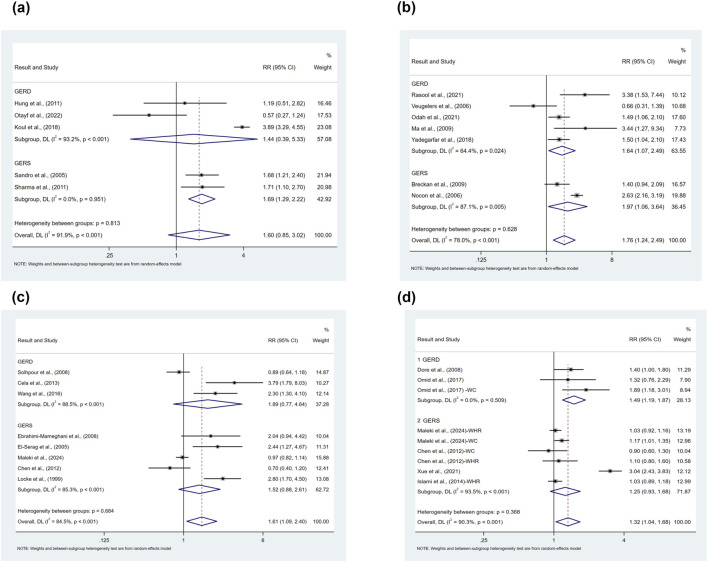
**(a)** Forest plot to examine effect size and data dispersion in the publications in obese and underweight groups. **(b)** Forest plot to examine effect size and data dispersion in the publications in obese and normal BMI groups. **(c)** Forest plot to examine effect size and data dispersion in the publications in obese and non-overweight groups. **(d)** Forest plot to examine effect size and data dispersion in the publications in obese and non-obese groups.

There were four categories of obesity analyzed, 1) obese vs. underweight population, 2) obese vs. normal BMI population, 3) obese vs. non-overweight population, and 4) obese vs. non-obese population. Our findings showed that obesity was associated with an elevated risk of prevalence of symptomatic GER and GERD compared to the underweight population (RR = 1.60, 95%CI 0.85–3.02; I^2^ 91.9%) as shown in Figure 7a. The results of the subgroup analyses suggested that publication year, income level, and sample size may be sources of heterogeneity, but meta-regression did not identify sources of heterogeneity. Due to the limited number of studies, subgroup analyses as well as publication bias analyses were not performed for confounders.

Obesity was associated with an elevated risk of prevalence of symptomatic GER and GERD compared with normal BMI population (RR = 1.76, 95%CI 1.24–2.49; I^2^ 78.0%), as shown in Figure 7b. The results of the subgroup analyses suggested that study type, study quality, and sample size may be sources of heterogeneity, but meta-regression did not identify sources of heterogeneity. Due to the limited number of studies, subgroup analyses as well as publication bias analyses were not performed for confounders.

Obesity was associated with an elevated risk of prevalence of symptomatic GER and GERD compared with the non-overweight population (RR = 1.61, 95%CI 1.09–2.40; I^2^ 84.5%), as shown in Figure 7c. The results of the subgroup analyses suggested that year of publication, level of income, and sample size might be a source of heterogeneity, but Meta-regression did not find a source of heterogeneity. Due to the limited number of studies, subgroup analyses as well as publication bias analyses were not performed for confounders.

In the comparison of the risk of prevalence of symptomatic GER and GERD in obese versus non-obese populations, we included diagnostic bases of obesity other than BMI mentioned in the previous study including Waist-to-Hip Ratio (WHR) and Waist Circumference (WC) for subgroup analysis. Obesity was associated with an elevated risk of prevalence of symptomatic GER and GERD compared with the non-obese population (RR = 1.32, 95%CI 1.04–1.68; I^2^ 90.3%), as shown in Figure 7d. Subgroup analyses did not identify factors contributing to heterogeneity, and the results of the meta-regression suggested that the diagnostic criteria for obesity might be a factor influencing the magnitude of heterogeneity (p = 0.015). For publication bias, the inverted funnel plot was symmetric (Supplementary Figure S3), which could be demonstrated by a non-significant Egger’s test (p = 0.909).

#### 4.2.5 Class Ⅰ obese (BMI 30–34.9 kg/m^2^)

**FIGURE 8:**
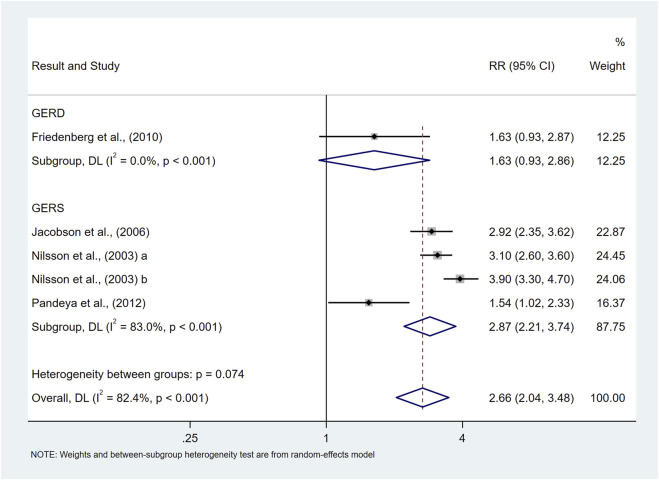
Forest plot to examine effect size and data dispersion in the publications in Class Ⅰ Obese and normal BMI groups.

The results of our study showed that class Ⅰ obese was associated with an elevated risk of prevalence of symptomatic GER and GERD (RR = 2.66, 95%CI 2.04–3.48; I^2^ 82.4%) compared to the normal BMI population, as shown in Figure 8. The results of the subgroup analyses suggested that the type of study, and the sample size may be the source of heterogeneity, and the results of the meta-regression suggested that the sample size may be a factor influencing the size of heterogeneity (p = 0.036), which is consistent with the results of subgroup analysis. Due to the limited number of studies, subgroup analyses as well as publication bias analyses were not performed for the confounders.

#### 4.2.6 Class II obese and above (BMI≥ 35 kg/m^2^)

**FIGURE 9:**
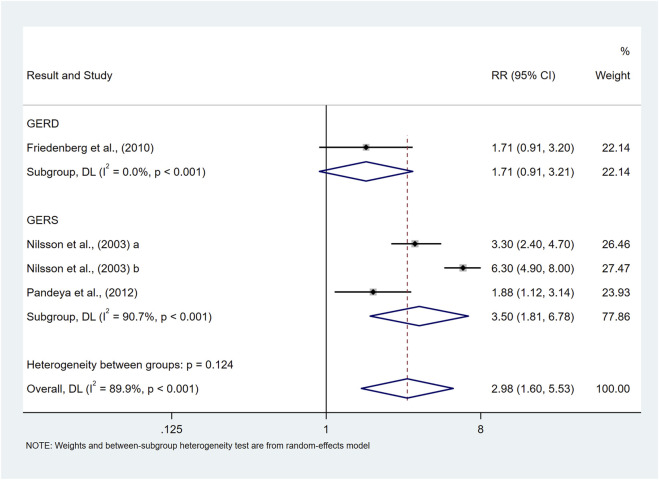
Forest plot to examine effect size and data dispersion in the publications in Class Ⅱ Obese and above and non-overweight groups.

Our study showed that class Ⅱ obese and above were associated with an elevated risk of prevalence of symptomatic GER and GERD (RR = 2.98, 95%CI 1.60–5.53; I^2^ 89.9%) compared to non-overweight populations, as shown in Figure 9. The results of subgroup analyses suggested that the type of study, and the sample size may be a source of heterogeneity, but meta-regression did not identify a source of heterogeneity. Due to the limited number of studies, subgroup analyses as well as publication bias analyses were not performed for confounders.

Due to the limited number of studies, subgroup analysis, meta-regression, and publication bias analysis were not conducted for the comparisons of the risk of symptomatic GER and GERD between the following groups: non-underweight versus underweight people (n = 1), class Ⅰ and Ⅱ obese versus underweight people (n = 1), class Ⅱ obese and above versus normal BMI people (n = 1), and class Ⅲ obese and above versus underweight people (n = 1) were not analyzed for subgroups, meta-regression and publication bias analysis.

### 4.3 Dose-response analysis

**FIGURE 10:**
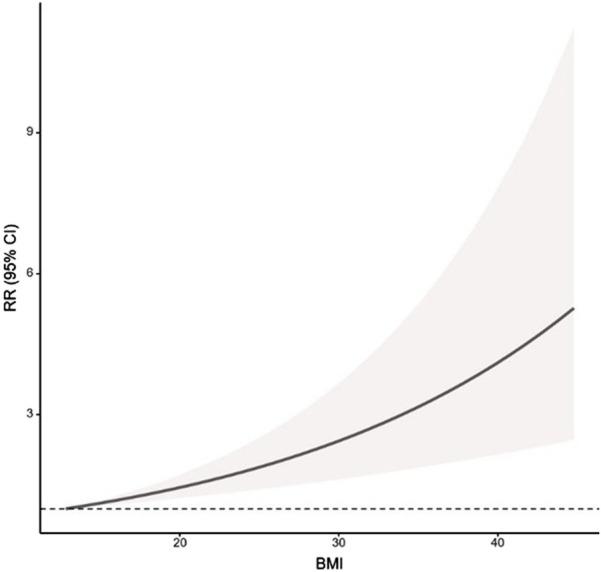
The linear dose response relationship between BMI and the prevalence of symptomatic GER and GERD. GERS = symptomatic gastroesophageal reflux disease; GERD = gastroesophageal reflux disease; RR = relative risk.

19 studies (Rasool et al., 2021; Islami et al., 2014; Dore et al., 2008; Baroni et al., 2023; Solhpour et al., 2008; Lin et al., 2019; Yadegarfar et al., 2018; Nilsson et al., 2003; Veugelers et al., 2006; El-Serag et al., 2005; Pandeya et al., 2012; Friedenberg et al., 2010; Ghoshal et al., 2021; Sadeghi et al., 2024; Wang et al., 2016; Hollenz et al., 2002; Bert et al., 2021; Hung et al., 2011; Ma et al., 2009) that reported 3 or more BMI subgroups (20 data sets in total) with a total of 268,151 subjects (50,756 patients with GERD and symptomatic GER) were included in the study for dose-response analysis. The analysis found a linear relationship between BMI and the prevalence of symptomatic GER and GERD (χ^2^ = 18.4628, p < 0.001) (Figure 10), with the dose-response relationship showing a positive monotonic curve shape. For every ad-ditional 10 kg/m^2^ of BMI, there was a 68% increase in the risk of disease prevalence (RR = 1.681, 95% CI 1.326–2.131).

## 5 Discussion

According to 2025 WHO World Obesity Atlas, lifestyle factors, including smoking, physical inactivity, poor diet, and overweight/obesity, are responsible for more than half of all premature deaths attributed to Non-Communicable Diseases (NCDs) globally, accounting for approximately 10.7 million deaths in 2021. The 2024 Global Burden of Disease (GBD) study indicated that 1.6 million (15%) of these premature deaths were specifically attributable to high BMI. Furthermore, in 2021, adults lost an estimated 161.1 million Disability-Adjusted Life Years (DALYs) due to NCDs influenced by known risk factors. Of these cumulative losses, 44.3 million (27%) were attributed to high BMI, as reported by the 2024 GBD study. Therefore, weight loss plays an important role in the prevention and treatment of many diseases. Previous studies on the relationship between BMI and GERD symptoms have shown (Jacobson et al., 2006) that increased BMI was positively associated with both the risk as well as the severity of GERD symptoms, and that there was a dose-dependent relationship between increased BMI and frequent GERD symptoms (Multivariable-adjusted trends p < 0.001). A large prospective cohort of 29,610 subjects (Ness-Jensen et al., 2013) found that weight loss was dose-dependently associated with a reduction in GERD symptoms and an increase in the success of anti-reflux medication. A decrease in BMI of more than 3.5 kg/m^2^ significantly reduced the number of patients with symptomatic GER who were not on medication or who were being treated with medication, suggesting that weight loss not only reduces the incidence of symptoms but also increases the se-verity of symptoms in symptomatic GER patients, and the efficacy is correlated with the magnitude of BMI reduction. Although the 2020 China GERD Expert Consensus (Chinese Society of Gastroenterology, 2020) and the American College of Gastroenterology (ACG) guidelines (Katz et al., 2022) recommend lifestyle modifications such as weight loss, smoking cessation, and elevation of the head of the bed to improve the GERD symptoms, these basic treatments are still easily ignored by clinicians.

This meta-analysis provides an overview of current relevant studies by examining the relationship between BMI and the prevalence of symptomatic GER and GERD. By integrating data from 43 medium and high-quality cross-sectional and case-control studies, our study provides strong evidence to support the notion that higher BMI levels significantly increase the risk of symptomatic GER and GERD. This observation remained stable regardless of study design (cross-sectional or case-control studies), diagnostic method (questionnaires, experts, authoritative consensus, 24-h esophageal pH/impedance monitoring), and clinical outcome (symptomatic GER or GERD). Specifically, we found that the RR of symptomatic GER or GERD was 2.041/1.373 in individuals with higher BMI compared with those with the lowest BMI levels. The findings suggested that BMI may increase the risk of symptomatic GER and GERD, but with greater heterogeneity between studies. Meanwhile, being overweight (BMI≥25 kg/m^2^), as an important inflection point for the risk of the disease, can be considered as a threshold for the initial screening of GERD and be included in the routine physical examination questionnaire, to establish a system of “screening - monitoring - intervention of the risk of GERD in overweight period,” which can move forward the preventive gateway, and promote the change from treatment of the disease to the risk interception.

Based on these findings, we investigated the relationship between different BMI levels and the prevalence of symptomatic GER and GERD and found that there was a significant positive linear correlation between BMI and the risk of symptomatic GER and GERD, with a 68% increase in the risk of disease for each increase in BMI of 10 kg/m^2^. This robust trend strongly suggests that the pathophysiological mechanisms linking obesity to GERD are not merely threshold-based but are continuously aggravated by increasing adiposity. The mechanisms can be broadly categorized into the following pathways (Vakil et al., 2007): Inducing anatomical alterations in the gastroesophageal junction through multiple mechanical effects: Obesity leads to pathologically elevated intra-abdominal pressure, hiatal hernia formation, and reduced LES tone. These changes collectively reverse pressure gradients, disrupting anatomical structures and directly impairing the anti-reflux barrier function, which exacerbate gastric reflux, thereby promoting the onset and progression of GERD (Eusebi et al., 2018). Driving GERD pathogenesis via adipocyte cytokines: Studies demonstrate a significant association between low adiponectin levels and high incidence rates of erosive esophagitis and Barrett’s esophagus (Thomas et al., 2016), while serum leptin levels correlate positively with GERD onset (Engineer et al., 2012). Obesity-related GERD patients commonly exhibit elevated leptin levels and leptin receptor (ObR) downregulation, a state of leptin resistance. This imbalance exacerbates reflux symptoms, intensifies mucosal damage, and correlates closely with endoscopic lesion severity. As demonstrated above, obesity-associated adipokines adiponectin and leptin jointly mediate the inflammatory injury and carcinogenic progression of GERD. Their imbalance constitutes a crucial molecular basis for obesity-related malignant transformation in GERD (El-Serag et al., 2014). Driving GERD pathogenesis through inflammation: Chronic low-grade metabolic inflammation induced by obesity is fueled by excessive caloric intake, leading to sustained release of pro-inflammatory factors from metabolic cells in adipose tissue, liver, and elsewhere. This triggers systemic inflammatory spread (Rieder et al., 2010). Chronic inflammation can induce multi-organ fibrosis, including in the esophagus, and this mechanism has been identified in the gastroesophageal mucosa of GERD patients (Garcia et al., 2014). Therefore, the observed dose-response relationship is not merely a statistical correlation but biologically plausible. It is driven by the synergistic effects of escalating mechanical stress from visceral fat accumulation and progressive inflammatory burden from dysregulated adipokines and inflammatory factors, underscoring the critical importance of weight management as a primary strategy for GERD prevention and control.

Our study provides a nuanced understanding of the relationship between BMI and GERD risk through the application of both categorical and dose-response analyses. The finding that BMI ≥25 kg/m^2^ serves as a significant inflection point is highly relevant for public health strategies and clinical screening, as it offers a clear, actionable threshold for initial risk stratification. This categorical increase in risk should not be misinterpreted as evidence against a continuous relationship. On the contrary, our dose-response meta-analysis, which is the first to quantitatively model this relationship for GERD, confirms a steady, linear increase in risk with rising BMI. This linear trend is consistent with the biological plausibility that even incremental increases in body weight can exacerbate the pathophysiological mechanisms driving GERD. Thus, the categorical and dose-response findings are synergistic. The inflection point at BMI ≥25 kg/m^2^ has pragmatic utility for identifying at-risk populations and setting intervention priorities, while the linear relationship underscores the importance of weight management across the entire population, including those within the normal and overweight ranges, to mitigate GERD risk progressively.

To investigate the specific causes of this heterogeneity, we conducted subgroup analyses. Results showed that publication year (p = 0.001), country/region (p = 0.035), study outcome (p = 0.014), other obesity indicators (p < 0.001), sample size (p = 0.038), and the confounding factors smoking (p = 0.035), education level (p = 0.003), dietary habits (p = 0.030), and medication history (p = 0.003) suggested these factors may significantly influence the pooled effect size. However, only country/region, other obesity indicators, and education level showed a significant reduction in heterogeneity after stratification, suggesting they may be sources of study heterogeneity. Meta-regression results indicated that the year of publication of the article, the category of clinical outcomes, dietary habits and medication history among the confounders were significantly associated with heterogeneity. This may be related to the increased sensitivity of GERD diagnostic criteria (e.g., widespread use of high-resolution esophageal manometry) in recent years and differences in pathomechanisms between GERD and symptomatic GER, while studies that did not control for dietary habits (e.g., high-fat intake) may have underestimated the independent effect of obesity, and studies that were not corrected for medication history (e.g., proton-pump inhibitor use) may have confounded the association between obesity and gastric acid secretion. Heterogeneity associated with countries/regions may stem from differences in dietary habits, lifestyles, and genetic susceptibility across countries/regions. For instance, the typical Western diet high in fat and processed foods may synergistically exacerbate reflux in obese individuals, whereas traditional diets in certain Asian regions may offer protective effects (Gamba et al., 2015). Furthermore, fundamental differences exist between obesity diagnostic standards; for example, WHR may be more sensitive to abdominal obesity, while BMI tends to provide a more overall assessment (World Obesity Federation, 2025). Studies failing to adequately control these factors may overestimate or underestimate BMI’s true independent effect. The remaining observed heterogeneity is more likely to reflect genuine population-level differences rather than methodological bias or publication bias. Nevertheless, the results of the vast majority of subgroup analyses supported high BMI as a risk factor for GERD (p < 0.001), which suggests: first, obesity management should be included in GERD prevention strategies, especially in high-income countries (RR = 1.781) and high-risk groups such as European (RR = 2.046) and North American populations (RR = 2.003). This result is consistent with the World Obesity Atlas 2025 (World Obesity Federation, 2025) published by the Obesity Prevention and Control Society of Chinese Nutrition Society, which states that the prevalence of overweight populations (BMI 25∼< 30 kg/m^2^) has stabilized in high-income countries, whereas this trend has not yet been observed in countries at other income levels.

Despite the utility of BMI as a screening tool in identifying potentially obese populations, detecting obesity based on BMI alone may lead to less comprehensive findings as novel obesity indicators continue to emerge. When classifying and analyzing different BMI levels, we included WC, WHR and other obesity indicators in the comparison of disease risk between obese and non-obese populations, but the meta-regression results suggest that inconsistencies in the classification standards across the studies may have led to significant heterogeneity, suggesting that our standardized obesity assessment system may have become a prerequisite for accurate disease risk stratification. Several studies have investigated the effect of different obesity indicators on GERD, Sadafi et al. (2024) observed that visceral fat area (VFA) was significantly higher in patients with GERD than in non-GERD patients (126.01 vs. 121.60 cm^2^, p = 0.008), and percent body fat (PBF) was also significantly higher than that of non-GERD patients, which is statistically significant (p = 0.003); after adjusting for regression modeling, WHR significantly increased the risk of GERD (OR= 1.94, 95% CI: 1.12–5.23); whereas the study by Chen et al. (2012) showed that there was no significant relationship between BMI, WHR or WC and the occurrence of reflux symptoms. In order to more accurately assess the degree and type of obesity, the Lancet Consensus recommends referring to at least one body measure in addition to BMI, at least two body measures when BMI is not calculated, or a direct measurement of body fat to confirm body fat content and its distribution.

We acknowledge that WHO recommend lower BMI thresholds for defining overweight and obesity for Asian populations (e.g., overweight: 23.0–27.5 kg/m^2^, obesity: ≥27.5 kg/m^2^) (WHO Expert Consultation, 2004) due to differences in body composition and higher health risks at lower BMIs. However, as the vast majority of the original studies did not employ or report race- or region-specific BMI categories, a stratified analysis using these adjusted cut-offs was not feasible in the present study. Consequently, the use of universal WHO BMI cut-offs (e.g., overweight ≥25 kg/m^2^; obese ≥30 kg/m^2^) in our study, while necessary for consistency, may lead to a systematic underestimation of GERD risk in Asian and other specific ethnic groups. Future high-quality prospective studies should prioritize the application of ethnic-specific BMI classifications, which will be crucial for a more precise and clinically relevant risk stratification across diverse global populations. This approach will not only clarify the true magnitude of the association, but also inform the development of tailored public health interventions and clinical screening guidelines that are sensitive to ethnic differences.

Our study has the following strengths. First, to our knowledge, this is the first dose-response analysis to assess BMI and risk of GERD prevalence, and the first meta-analysis study to categorize the risk of GERD prevalence for different levels of BMI to facilitate evidence-based determination of disease risk thresholds. Second, for the selection of GERD patients for the study, we used strict inclusion criteria, such as the use of a validated and reliable authoritative GERD diagnostic questionnaire. Third, we performed confounders analyses to identify factors affecting the prevalence of symp-tomatic GER and GERD. Finally, we used different meta-analytic methods to explore the relationship between BMI and GERD risk, and all methods showed consistent results that higher BMI was associated with higher GERD risk.

Despite the novelty and significance of our analysis, its limitations must be recognized. First, although our subgroup analysis incorporated WHR and WC as alternative obesity indicators, the limited number of studies reporting these measures prevented comprehensive stratified analyses, and more comprehensive diagnostic methods such as WHR and relative fat mass (RFM) were not considered. Body fat distribution measurements and individual health status should be combined to overcome the shortcomings of BMI as a single indicator to provide more scientific diagnosis and management for obese patients. Second, the large heterogeneity of the study’s combined effects may affect the reliability of the findings, but the study performed exhaustive subgroup analyses and meta-regression to explore the sources of heterogeneity. In addition, obesity-related surgery may lead to an elevated risk of symptomatic GER and GERD, which was not considered in our study. Fourth, the diagnostic criteria for GERD showed variability across the included studies. Notably, for the analysis of studies using 24-h pH monitoring, we applied a uniform AET threshold of >4.2% to maintain consistency. While this cut-off is well-established in historical literature, it does not reflect the more stringent criteria (e.g., AET >6%) recommended in contemporary consensus guidelines like the 2018 Lyon Consensus (Gyawali et al., 2018). This methodological choice was necessary to accommodate older studies and maximize data pooling, but it may have influenced the generalizability of our findings to current clinical practice, where diagnostic thresholds are higher and more refined. Finally, since both GERD and obesity are progressive processes, defining their temporal relationship may be challenging. The cross-sectional nature of the studies included in our result meant that we were unable to establish a causal relationship between BMI increase and GERD, more relevant prospective cohort designs should be conducted in the future to track the dose-effect relationship between dynamic changes in body weight and the onset of GERD to establish a more rigorous and standardized baseline for the prevalence and duration of GERD and obesity, which would help to further elucidate the targeted interventions’ potential impact.

Although our meta-analysis supports the idea that obesity increases the risk of developing GERD, the exact mechanism of this effect remains unclear, and various biochemical and psychosocial factors, in addition to anatomical and physiological structural changes due to obesity, may be responsible for this relationship. The necessity of conducting medical imaging and biomarker (e.g., adiponectin, IL-6, etc.) related studies to explore the pathological pathways of obesity-related inflammation and esophageal mucosal injury has been proven in practice. Besides, future studies should consider objective diagnostic methods (e.g., 24-h esophageal pH/impedance monitoring) to reduce inter-study variability and provide more reliable evidence for clinical guidelines.

## 6 Conclusion

In summary, our study provides evidence to support that higher levels of BMI increase the risk of GERD prevalence and that there is a significant positive linear association between BMI and the risk of GERD prevalence. Overweight (BMI ≥25 kg/m^2^) serves as an important inflection point for disease risk. These findings emphasize the need for further research on this relationship, and more prospective cohort study designs with objective diagnostic methods and comprehensive observational indicators are still needed in the future to explore the causal relationship between dynamic changes in BMI and the onset of GERD, as well as to explore the mechanisms.

## Data Availability

The original contributions presented in the study are included in the article/Supplementary Material, further inquiries can be directed to the corresponding authors.
