# Supplementary material for: Association between body mass index at different levels and risk of gastroesophageal reflux disease: a systematic review with dose-response meta-analysis
Source: Front Physiol. 2025 Nov 26;16:1675457. doi: 10.3389/fphys.2025.1675457 (PMC12689342; doi:10.3389/fphys.2025.1675457)
Supplement: Supplementary file 1 [file DataSheet1.docx]

Supplementary Material

# Supplementary Data

The document of International Prospective Register of Systematic Reviews (PROSPERO: CRD42024563046).

# Supplementary Figures and Tables

##
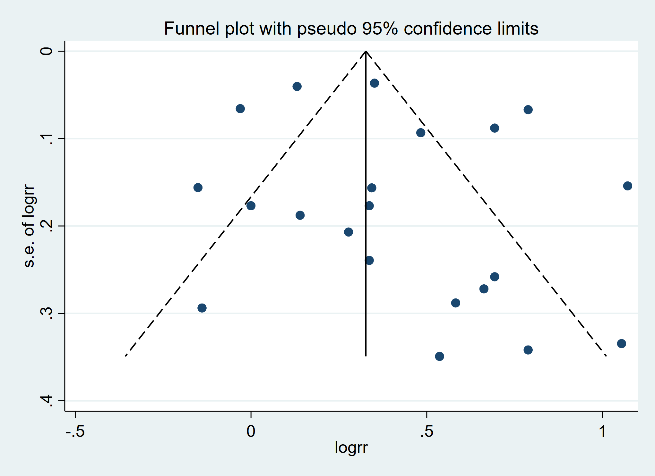
Supplementary Figures

**Supplementary Figure 1.** Funnel plot for publication bias in overweight and non-overweight groups.


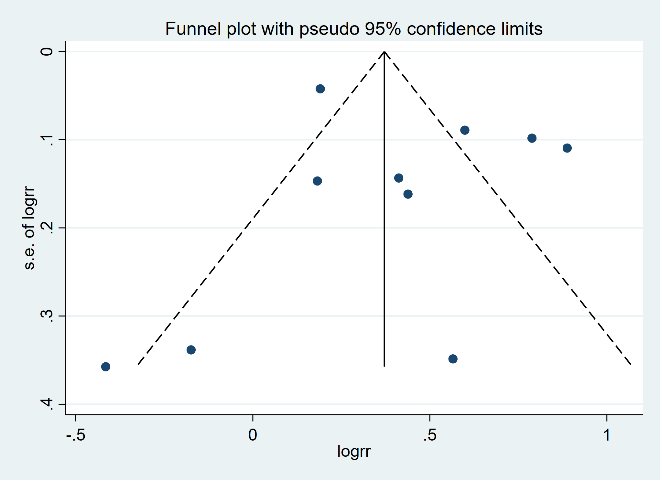


**Supplementary Figure 2.** Funnel plot for publication bias in over-weight and normal BMI groups.


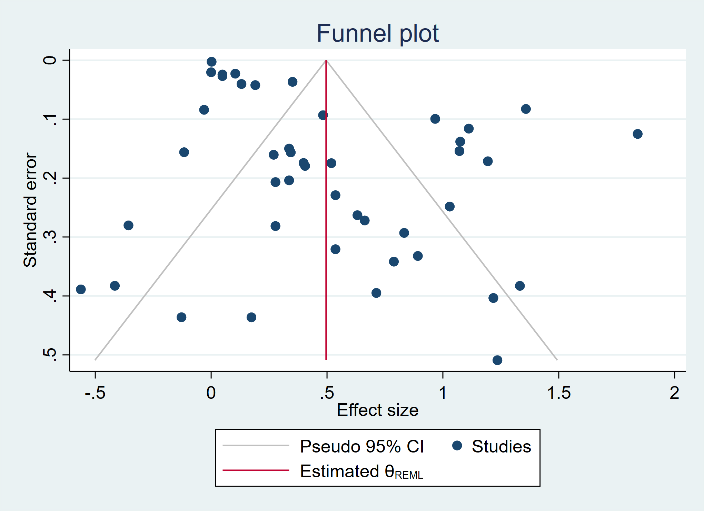

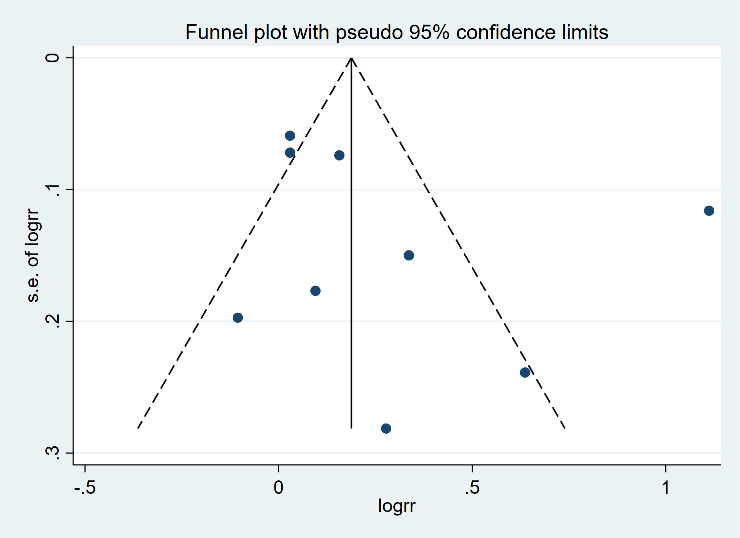
**Supplementary Figure 3.** Funnel plot for publication bias in obese and non-obese groups.


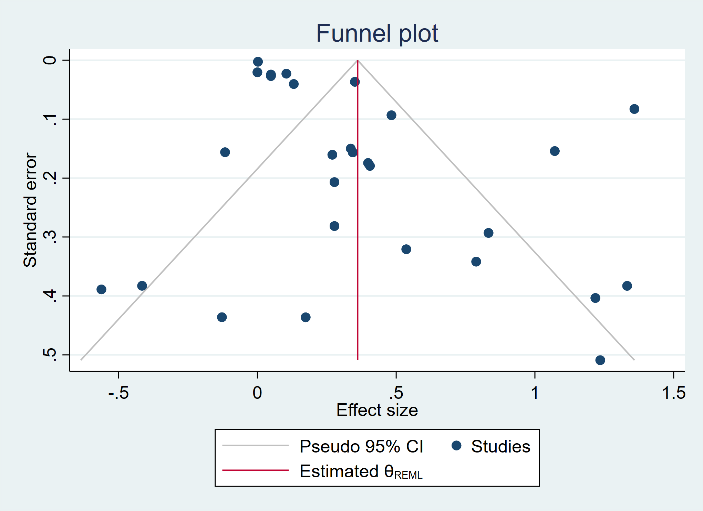
**Supplementary Figure 4.** Funnel plot for included studies of BMI and Risk of Symptomatic GERD/GERD under trim-and-fill method.

**Supplementary Figure 5.** Funnel plot for included studies of BMI and Risk of GERD under trim-and-fill method.

## Supplementary Tables

# Supplementary Table 1. Pubmed Search Strategy

| **Search** | **Query** |
| --- | --- |
| #1 | ("Gastroesophageal Reflux"[Mesh]) OR ((((((((((((((Gastroesophageal Reflux[Title/Abstract]) OR (GERD[Title/Abstract])) OR (Esophageal Reflux[Title/Abstract])) OR (Gastric Acid Reflux[Title/Abstract])) OR (Acid Reflux, Gastric[Title/Abstract])) OR (Reflux, Gastric Acid[Title/Abstract])) OR (Gastric Acid Reflux Disease[Title/Abstract])) OR (Gastro-Esophageal Reflux[Title/Abstract])) OR (Gastro Esophageal Reflux[Title/Abstract])) OR (Reflux, Gastro-Esophageal[Title/Abstract])) OR (Gastro-oesophageal Reflux[Title/Abstract])) OR (Gastro oesophageal Reflux[Title/Abstract])) OR (Reflux, Gastro-oesophageal[Title/Abstract])) OR (Reflux, Gastroesophageal[Title/Abstract])) |
| #2 | ("Heartburn"[Mesh]) OR (((Heartburn[Title/Abstract]) OR (Pyrosis[Title/Abstract])) OR (Pyroses[Title/Abstract])) |
| #3 | ("Esophageal Sphincter, Lower"[Mesh]) OR ((((((esophageal sphincter, lower[Title/Abstract]) OR (Sphincter, Lower Esophageal[Title/Abstract])) OR (Gastroesophageal Sphincter[Title/Abstract])) OR (Gastroesophageal Sphincters[Title/Abstract])) OR (Sphincter, Gastroesophageal[Title/Abstract])) OR (Lower Esophageal Sphincter[Title/Abstract])) |
| #4 | ("Esophageal pH Monitoring"[Mesh]) OR (((((((((esophageal ph monitoring[Title/Abstract]) OR (pH Monitoring, Esophageal[Title/Abstract])) OR (Esophageal pH Recording[Title/Abstract])) OR (pH Recording, Esophageal[Title/Abstract])) OR (Recording, Esophageal pH[Title/Abstract])) OR (Monitoring, Esophageal pH[Title/Abstract])) OR (Ambulatory Esophageal pH Monitoring[Title/Abstract])) OR (Ambulatory 24-hour Esophageal pH Monitoring[Title/Abstract])) OR (Ambulatory 24 hour Esophageal pH Monitoring[Title/Abstract])) |
| #5 | ("Laryngopharyngeal Reflux"[Mesh]) OR ((((((((((laryngopharyngeal reflux[Title/Abstract]) OR (Reflux, Laryngopharyngeal[Title/Abstract])) OR (Regurgitation, Gastric[Title/Abstract])) OR (Gastric Regurgitation[Title/Abstract])) OR (Supraesophageal Gastric Reflux (SEGR[Title/Abstract]))) OR (Gastric Reflux, Supraesophageal (SEGR[Title/Abstract]))) OR (Reflux, Supraesophageal Gastric (SEGR[Title/Abstract]))) OR (Supraesophageal Gastric Reflux[Title/Abstract])) OR (Gastric Reflux, Supraesophageal[Title/Abstract])) OR (Reflux, Supraesophageal Gastric[Title/Abstract])) |
| #6 | ("Obesity"[Mesh]) OR ((Obesity[Title/Abstract]) OR (Obese[Title/Abstract])) |
| #7 | ("Overweight"[Mesh]) OR (Overweight[Title/Abstract]) |
| #8 | ("Body Mass Index"[Mesh]) OR (((((((Body Mass Index[Title/Abstract]) OR (BMI[Title/Abstract])) OR (Index, Body Mass[Title/Abstract])) OR (Quetelet's Index[Title/Abstract])) OR (Quetelets Index[Title/Abstract])) OR (Quetelet Index[Title/Abstract])) OR (Index, Quetelet[Title/Abstract])) |
| #9 | ("Cohort Studies"[Mesh]) OR ((((Cohort Studies[Title/Abstract]) OR (Cohort Study[Title/Abstract])) OR (Studies, Cohort[Title/Abstract])) OR (Study, Cohort[Title/Abstract])) |
| #10 | ("Cross-Sectional Studies"[Mesh]) OR (((((Cross-Sectional Studies[Title/Abstract]) OR (Cross Sectional Studies[Title/Abstract])) OR (Cross-Sectional Study[Title/Abstract])) OR (Studies, Cross-Sectional[Title/Abstract])) OR (Study, Cross-Sectional[Title/Abstract])) |
| #11 | ("Case-Control Studies"[Mesh]) OR (((((((Case-Control Studies[Title/Abstract]) OR (Case-Control Study[Title/Abstract])) OR (Studies, Case-Control[Title/Abstract])) OR (Study, Case-Control[Title/Abstract])) OR (Case-Comparison Studies[Title/Abstract])) OR (Case Comparison Studies[Title/Abstract])) OR (Case-Comparison Study[Title/Abstract])) |
| #12 | #1 OR #2 OR #3 OR #4 OR #5 |
| #13 | #6 OR #7 OR #8 |
| #14 | #9 OR #10 OR #11 |
| #15 | #12 AND #13 AND #14 |

**Supplementary Table 2. Embase Search Strategy**

| **Search** | **Query** |
| --- | --- |
| #1 | 'gastroesophageal reflux'/exp OR 'gastroesophageal reflux' |
| #2 | 'gerd':ab,ti OR 'esophageal reflux':ab,ti OR 'gastric acid reflux':ab,ti OR 'acid reflux, gastric':ab,ti OR 'reflux, gastric acid':ab,ti OR 'gastric acid reflux disease':ab,ti OR 'gastro-esophageal reflux':ab,ti OR 'gastro esophageal reflux':ab,ti OR 'reflux, gastro-esophageal':ab,ti OR 'gastro-oesophageal reflux':ab,ti OR 'gastro oesophageal reflux':ab,ti OR 'reflux, gastro-oesophageal':ab,ti OR 'reflux, gastroesophageal':ab,ti |
| #3 | #1 OR #2 |
| #4 | 'heartburn'/exp OR 'heartburn' |
| #5 | 'pyrosis':ab,ti OR 'pyroses':ab,ti |
| #6 | #4 OR #5 |
| #7 | 'lower esophagus sphincter'/exp OR 'lower esophagus sphincter' |
| #8 | 'sphincter, lower esophageal':ab,ti OR 'gastroesophageal sphincter':ab,ti OR 'gastroesophageal sphincters':ab,ti OR 'sphincter, gastroesophageal':ab,ti OR 'lower esophageal sphincter':ab,ti |
| #9 | #7 OR #8 |
| #10 | 'esophageal ph monitoring'/exp OR 'esophageal ph monitoring' |
| #11 | 'ph monitoring, esophageal':ab,ti OR 'esophageal ph recording':ab,ti OR 'ph recording, esophageal':ab,ti OR 'recording, esophageal ph':ab,ti OR 'monitoring, esophageal ph':ab,ti OR 'ambulatory esophageal ph monitoring':ab,ti OR 'ambulatory 24-hour esophageal ph monitoring':ab,ti OR 'ambulatory 24 hour esophageal ph monitoring':ab,ti |
| #12 | #10 OR #11 |
| #13 | 'laryngopharyngeal reflux'/exp OR 'laryngopharyngeal reflux' |
| #14 | 'reflux, laryngopharyngeal':ab,ti OR 'regurgitation, gastric':ab,ti OR 'gastric regurgitation':ab,ti OR 'supraesophageal gastric reflux (segr)':ab,ti OR 'gastric reflux, supraesophageal (segr)':ab,ti OR 'reflux, supraesophageal gastric (segr)':ab,ti OR 'supraesophageal gastric reflux':ab,ti OR 'gastric reflux, supraesophageal':ab,ti OR 'reflux, supraesophageal gastric':ab,ti |
| #15 | #13 OR #14 |
| #16 | 'obesity'/exp OR obesity |
| #17 | 'obese':ab,ti |
| #18 | #16 OR #17 |
| #19 | overweight'/exp OR overweight |
| #20 | 'overweight':ab,ti |
| #21 | #19 OR #20 |
| #22 | 'body mass'/exp OR 'body mass' |
| #23 | 'body mass index':ab,ti OR 'bmi':ab,ti OR 'index, body mass':ab,ti OR 'quetelets index':ab,ti OR 'quetelet index':ab,ti OR 'index, quetelet':ab,ti |
| #24 | #22 OR #23 |
| #25 | 'cohort studies':ab,ti OR 'cohort study':ab,ti OR 'studies, cohort':ab,ti OR 'study, cohort':ab,ti OR 'cross-sectional studies':ab,ti OR 'cross sectional studies':ab,ti OR 'cross-sectional study studies, cross-sectional':ab,ti OR 'study, cross-sectional':ab,ti OR 'case-control studies':ab,ti OR 'case-control study':ab,ti OR 'studies, case-control':ab,ti OR 'study, case-control':ab,ti OR 'case-comparison studies':ab,ti OR 'case comparison studies':ab,ti OR 'case-comparison study':ab,ti |
| #26 | #3 OR #6 OR #9 OR #12 OR #15 |
| #27 | #18 OR #21 OR #24 |

**Supplementary Table 3. Cochrane library Search Strategy**

| **Search** | **Query** |
| --- | --- |
| #1 | MeSH descriptor: [Gastroesophageal Reflux] explode all trees |
| #2 | (Gastroesophageal Reflux or GERD or Esophageal Reflux or Gastric Acid Reflux or Acid Reflux, Gastric or Reflux, Gastric Acid or Gastric Acid Reflux Disease or Gastro-Esophageal Reflux or Gastro Esophageal Reflux or Reflux, Gastro-Esophageal or Gastro-oesophageal Reflux or Gastro oesophageal Reflux or Reflux, Gastro-oesophageal or Reflux, Gastroesophageal):ti,ab,kw (Word variations have been searched) |
| #3 | #1 or #2 |
| #4 | MeSH descriptor: [Heartburn] explode all trees |
| #5 | (Heartburn or Pyrosis or Pyroses):ti,ab,kw (Word variations have been searched) |
| #6 | #4 or #5 |
| #7 | MeSH descriptor: [Esophageal Sphincter, Lower] explode all trees |
| #8 | (esophageal sphincter, lower or Sphincter, Lower Esophageal or Gastroesophageal Sphincter or Gastroesophageal Sphincters or Sphincter, Gastroesophageal or Lower Esophageal Sphincter):ti,ab,kw (Word variations have been searched) |
| #9 | #7 or #8 |
| #10 | MeSH descriptor: [Esophageal pH Monitoring] explode all trees |
| #11 | (esophageal ph monitoring or pH Monitoring, Esophageal or Esophageal pH Recording or pH Recording, Esophageal or Recording, Esophageal pH or Monitoring, Esophageal pH):ti,ab,kw (Word variations have been searched) |
| #12 | #10 or #11 |
| #13 | MeSH descriptor: [Laryngopharyngeal Reflux] explode all trees |
| #14 | (laryngopharyngeal reflux or Reflux, Laryngopharyngeal or Regurgitation, Gastric or Gastric Regurgitation or Supraesophageal Gastric Reflux (SEGR) or Gastric Reflux, Supraesophageal (SEGR) or Reflux, Supraesophageal Gastric (SEGR) or Supraesophageal Gastric Reflux or Gastric Reflux, Supraesophageal or Reflux, Supraesophageal Gastric):ti,ab,kw (Word variations have been searched) |
| #15 | #13 or #14 |
| #16 | MeSH descriptor: [Obesity] explode all trees |
| #17 | (Obesity or Obese):ti,ab,kw (Word variations have been searched) |
| #18 | #16 or #17 |
| #19 | MeSH descriptor: [Overweight] explode all trees |
| #20 | (Overweight):ti,ab,kw (Word variations have been searched) |
| #21 | #19 or #20 |
| #22 | MeSH descriptor: [Body Mass Index] explode all trees |
| #23 | (Body Mass Index or BMI or Index, Body Mass or Quetelet's Index or Quetelets Index or Quetelet Index or Index, Quetelet):ti,ab,kw (Word variations have been searched) |
| #24 | #22 or #23 |
| #25 | #3 or #6 or #9 or #12 or #15 |
| #26 | #18 or #21 or #24 |
| #27 | #25 and #26 |

**Supplementary Table 4. Web of science Search Strategy**

| **Search** | **Query** |
| --- | --- |
| #1 | Gastroesophageal Reflux (Topic) or GERD (Topic) or Esophageal Reflux (Topic) or Gastric Acid Reflux (Topic) or Acid Reflux, Gastric (Topic) or Reflux, Gastric Acid (Topic) or Gastric Acid Reflux Disease (Topic) or Gastro-Esophageal Reflux (Topic) or Gastro Esophageal Reflux (Topic) or Reflux, Gastro-Esophageal (Topic) or Gastro-oesophageal Reflux (Topic) or Gastro oesophageal Reflux (Topic) or Reflux, Gastro-oesophageal (Topic) or Reflux, Gastroesophageal (Topic) and Preprint Citation Index (Exclude – Database) |
| #2 | Heartburn (Topic) or Pyrosis (Topic) or Pyroses (Topic) and Preprint Citation Index (Exclude – Database) |
| #3 | esophageal sphincter, lower (Topic) or Sphincter, Lower Esophageal (Topic) or Gastroesophageal Sphincter (Topic) or Gastroesophageal Sphincters (Topic) or Sphincter, Gastroesophageal (Topic) or Lower Esophageal Sphincter (Topic) and Preprint Citation Index (Exclude – Database) |
| #4 | esophageal ph monitoring (Topic) or pH Monitoring, Esophageal (Topic) or Esophageal pH Recording (Topic) or pH Recording, Esophageal (Topic) or Recording, Esophageal pH (Topic) or Monitoring, Esophageal pH (Topic) or Ambulatory Esophageal pH Monitoring (Topic) or Ambulatory 24-hour Esophageal pH Monitoring (Topic) or Ambulatory 24 hour Esophageal pH Monitoring (Topic) and Preprint Citation Index (Exclude – Database) |
| #5 | laryngopharyngeal reflux (Topic) or Reflux, Laryngopharyngeal (Topic) or Regurgitation, Gastric (Topic) or Gastric Regurgitation (Topic) or Supraesophageal Gastric Reflux (SEGR) (Topic) or Gastric Reflux, Supraesophageal (SEGR) (Topic) or Reflux, Supraesophageal Gastric (SEGR) (Topic) or Supraesophageal Gastric Reflux (Topic) or Gastric Reflux, Supraesophageal (Topic) or Gastric Reflux, Supraesophageal (Topic) and Preprint Citation Index (Exclude – Database) |
| #6 | Obesity (Topic) or Obese (Topic) or Overweight (Topic) or Body Mass Index (Topic) or BMI (Topic) or Index, Body Mass (Topic) or Quetelet's Index (Topic) or Quetelets Index (Topic) or Quetelet Index (Topic) or Index, Quetelet (Topic) and Preprint Citation Index (Exclude – Database) |
| #7 | #1 OR #2 OR #3 OR #5 OR #4 and Preprint Citation Index (Exclude – Database) |
| #8 | Cohort Studies (Topic) or Cohort Study (Topic) or Studies, Cohort (Topic) or Study, Cohort (Topic) or Cross-Sectional Studies (Topic) or Cross Sectional Studies (Topic) or Cross-Sectional Study (Topic) or Studies, Cross-Sectional (Topic) or Study, Cross-Sectional (Topic) or Case-Control Studies (Topic) or Case-Control Study (Topic) or Studies, Case-Control (Topic) or Study, Case-Control (Topic) or Case-Comparison Studies (Topic) or Case Comparison Studies (Topic) or Case-Comparison Study (Topic) and Preprint Citation Index (Exclude – Database) |
| #9 | #6 AND #7 AND #8 and Preprint Citation Index (Exclude – Database) |

**Supplementary Table 5. PECOS Framework**

Study Eligibility Criteria

To align with the goals of our meta-analysis, we have devised a 'PECOS' framework, which stands for 'Population, Exposure, Comparison, Outcome, and Study.'

| Item | Definition |
| --- | --- |
| Population | Patients with GERS or GERD |
| Exposure | Underweight, overweight, and obesity assessed by calculating BMI |
| Comparison | Normal BMI |
| Outcome | Risk of GERS or GERD |
| Study | Observational designs (cohort, case-control, cross-sectional) |

GERS, symptomatic gastroesophageal reflux disease; GERD, gastroesophageal reflux disease; BMI, body mass index.

**Supplementary Table 6. Result of the quality assessment using the AHRQ**

| Author and year | Q1 | Q2 | Q3 | Q4 | Q5 | Q6 | Q7 | Q8 | Q9 | Q10 | Q11 | Score | Quality |
| --- | --- | --- | --- | --- | --- | --- | --- | --- | --- | --- | --- | --- | --- |
| Sandro et al., (2005) | Yes | Yes | Yes | No | Yes | Yes | Yes | Yes | No | Yes | No | 8 | High |
| Hollenz et al., (2002) | Yes | Yes | No | Yes | Yes | No | Yes | No | No | Yes | No | 6 | Median |
| Chen et al., (2021) | Yes | Yes | Yes | No | No | Yes | Yes | Yes | No | Yes | No | 7 | Median |
| Rasool et al., (2021) | Yes | Yes | Yes | No | No | Yes | Yes | Yes | No | Yes | No | 7 | Median |
| Jacobson et al., (2006) | Yes | Yes | Yes | No | No | Yes | Yes | Yes | No | Yes | No | 7 | Median |
| Watanabe et al., (2003) | Yes | Yes | Yes | Yes | No | Yes | Yes | Yes | No | Yes | No | 8 | High |
| Omid et al., (2017) | Yes | Yes | Yes | Yes | No | Yes | Yes | Yes | No | Yes | No | 8 | High |
| Baroni et al., (2023) | Yes | Yes | Yes | Unclear | Yes | Yes | Yes | Yes | No | Yes | No | 8 | High |
| Solhpour et al., (2008) | Yes | Yes | Yes | No | Yes | Yes | Yes | Yes | No | Yes | No | 8 | High |
| Rosaida et al., (2004) | Yes | Yes | Yes | Yes | Yes | Yes | Yes | Yes | No | Yes | No | 9 | High |
| Chen et al., (2024) | Yes | Yes | No | Unclear | No | Yes | Yes | Yes | No | Yes | No | 6 | Median |
| Tong et al., (2024) | Yes | Yes | Yes | No | No | Yes | Yes | Yes | No | Yes | No | 7 | Median |
| Lin et al., (2019) | Yes | Yes | Yes | No | No | Yes | Yes | Yes | No | Yes | No | 7 | Median |
| Cela et al., (2013) | Yes | Yes | Yes | No | No | Yes | No | Yes | No | Yes | No | 6 | Median |
| Wenzl et al., (2021) | Yes | No | No | No | No | Yes | No | Yes | No | Yes | No | 4 | Median |
| El-Serag et al., (2005) | Yes | Yes | No | No | Yes | Yes | Yes | Yes | Yes | Yes | No | 8 | High |
| Pandeya et al., (2012) | Yes | Yes | Yes | No | No | Yes | Yes | Yes | Yes | Yes | No | 8 | High |
| Friedenberg et al., (2010) | Yes | Yes | No | No | No | Yes | Yes | Yes | Yes | Yes | No | 7 | Median |
| Ghoshal et al., (2021) | Yes | Yes | Yes | Yes | No | Yes | No | No | No | Yes | No | 6 | Median |
| Sadeghi et al., (2024) | Yes | Yes | Yes | No | No | Yes | Yes | Yes | Yes | Yes | No | 8 | High |
| Maleki et al., (2024) | Yes | Yes | No | No | No | Yes | No | Yes | No | Yes | No | 5 | Median |
| Chen et al., (2012) | Yes | Yes | Yes | No | No | Yes | Yes | Yes | Yes | Yes | No | 8 | High |
| Odah et al., (2021) | Yes | Yes | Yes | No | No | Yes | No | No | No | Yes | No | 5 | Median |
| Wang et al., (2016) | Yes | Yes | Yes | No | No | Yes | No | Yes | No | Yes | No | 6 | Median |
| Hung et al., (2011) | Yes | Yes | Yes | Unclear | No | Yes | Yes | No | No | Yes | No | 6 | Median |
| Bert et al., (2021) | Yes | Yes | Yes | Unclear | No | Yes | No | Yes | Yes | Yes | No | 7 | Median |
| Ma et al., (2009) | Yes | Yes | No | No | No | Yes | No | Yes | No | Yes | No | 5 | Median |
| Liu et al., (2023) | Yes | Yes | Yes | No | No | Yes | No | Yes | No | Yes | No | 6 | Median |
| Sharma et al., (2011) | Yes | Yes | Yes | Yes | No | Yes | Yes | Yes | No | Yes | No | 8 | High |
| Locke et al., (1999) | Yes | Yes | Yes | No | Yes | Yes | Yes | Yes | No | Yes | No | 8 | High |
| Sadafi et al., (2024) | Yes | Yes | Yes | No | No | Yes | Yes | Yes | No | Yes | No | 7 | Median |
| Xue et al., (2021) | Yes | Yes | Yes | Yes | No | Yes | Yes | Yes | No | Yes | No | 8 | High |
| Abed et al., (2024) | Yes | Yes | Yes | No | No | Yes | Yes | Yes | No | Yes | No | 7 | Median |
| Breckan et al., (2009) | Yes | Yes | Yes | No | No | Yes | Yes | Yes | No | Yes | No | 7 | Median |
| Yadegarfar et al., (2008) | Yes | Yes | Yes | No | No | Yes | Yes | No | No | Yes | No | 6 | Median |
| Otayf et al., (2022) | Yes | Yes | Yes | No | No | Yes | No | No | No | Yes | No | 5 | Median |
| Nocon et al., (2006) | Yes | Yes | No | No | No | Yes | No | Yes | No | Yes | No | 5 | Median |
| Koul et al., (2018) | Yes | Yes | Yes | No | No | Yes | No | No | No | Yes | No | 5 | Median |
| Islami et al., (2014) | Yes | Yes | Yes | No | No | Yes | No | Yes | Yes | Yes | No | 7 | Median |

**Supplementary Table 7. Result of the quality assessment using the NOS**

| **Author and year** | **Q1** | **Q2** | **Q3** | **Q4** | **Q5** | **Q6** | **Q7** | **Q8** | **Score** | **Quality** |
| --- | --- | --- | --- | --- | --- | --- | --- | --- | --- | --- |
| Dore et al., (2008) | 1 | 1 | 1 | 1 | 1 | 1 | 1 | 1 | 8 | High |
| Ebrahimi-Mameghani et al., (2008) | 1 | 1 | 0 | 1 | 1 | 1 | 1 | 1 | 7 | High |
| Nilsson et al., (2003) | 1 | 1 | 1 | 1 | 1 | 1 | 1 | 0 | 7 | High |
| Veugelers et al., (2006) | 1 | 1 | 0 | 1 | 1 | 1 | 1 | 1 | 7 | High |

**Supplementary Table 8. Summary of certainty of evidence as assessed with the GRADE approach**

| **Outcome** | **NO.of studies** | **Studies Design** | **Certainty Assessment** | | | | | **RR[95%]** | **Certainty** |
| --- | --- | --- | --- | --- | --- | --- | --- | --- | --- |
|  |  |  | Risk of Bias | Inconsistency | Indirectness | Imprecision | Other Considerations |  |  |
| Prognostic factor: Underweight (vs normal BMI) | | | | | | | | | |
| GERD risk increase | 6 | case-control or cross-sectional study | not serious | not serious | not serious | not serious | Dose response gradient a | 0.90(0.71,1.15) | ⨁⨁⨁◯  MODERATEE |
| Prognostic factor: Normal BMI (vs underweight) | | | | | | | | | |
| GERD risk increase | 5 | case-control or cross-sectional study | not serious | not serious | not serious | serious | Dose response gradient a | 1.14(0.91,1.43) | ⨁⨁◯◯  LOW |
| Prognostic factor: Overweight (vs non-overweight) | | | | | | | | | |
| GERD risk increase | 21 | case-control or cross-sectional study | not serious | not serious | not serious | not serious | Dose response gradient a | 1.49(1.29,1.73) | ⨁⨁⨁◯  MODERATEE |
| Prognostic factor: Overweight (vs underweight) | | | | | | | | | |
| GERD risk increase | 5 | case-control or cross-sectional study | not serious | not serious | not serious | not serious | Dose response gradient a | 1.44(1.04，1.98) | ⨁⨁⨁◯  MODERATEE |
| Prognostic factor: Overweight (vs normal BMI ) | | | | | | | | | |
| GERD risk increase | 10 | case-control or cross-sectional study | not serious | not serious | not serious | not serious | Dose response gradient a | 1.51(1.21,1.89) | ⨁⨁⨁◯  MODERATEE |
| Prognostic factor: Obese (vs underweight) | | | | | | | | | |
| GERD risk increase | 5 | case-control or cross-sectional study | not serious | not serious | not serious | not serious | Dose response gradient a | 1.60(0.85,3.02) | ⨁⨁◯◯  LOW |
| Prognostic factor: Obese (vs normal BMI) | | | | | | | | | |
| GERD risk increase | 7 | case-control or cross-sectional study | not serious | not serious | not serious | not serious | Dose response gradient a | 1.76(1.24,2.49) | ⨁⨁⨁◯  MODERATEE |
| Prognostic factor: Obese (vs non-overweight ) | | | | | | | | | |
| GERD risk increase | 8 | case-control or cross-sectional study | not serious | not serious | not serious | not serious | Dose response gradient a | 1.61(1.09,2.40） | ⨁⨁⨁◯  MODERATEE |
| Prognostic factor: Obese (vs non-obese) | | | | | | | | | |
| GERD risk increase | 9 | case-control or cross-sectional study | not serious | not serious | not serious | not serious | Dose response gradient a | 1.36(1.08,1.71) | ⨁⨁⨁◯  MODERATEE |
| Prognostic factor: Class Ⅰ Obese (vs normal BMI) | | | | | | | | | |
| GERD risk increase | 5 | case-control or cross-sectional study | not serious | not serious | not serious | not serious | Dose response gradient a | 2.66(2.04,3.48) | ⨁⨁⨁◯  MODERATEE |
| Prognostic factor: Class Ⅱ Obese and above (vs non-overweight ) | | | | | | | | | |
| GERD risk increase | 4 | case-control or cross-sectional study | not serious | not serious | not serious | not serious | Dose response gradient a | 2.98(1.60,5.53） | ⨁⨁⨁◯  MODERATEE |

**Supplementary Table 9 Heterogeneity Metrics from Subgroup Analyses and Meta-regression of the Association Between BMI and GERD**

| Subgroup Variable | Subgroup Category | Heterogeneity | | p for between  group | Meta-regression |
| --- | --- | --- | --- | --- | --- |
|  |  | I^2^ | P |  |  |
| Publication year |  |  |  | 0.001 | 0.021 |
|  | before 2020 | 96.1% | ＜0.001 |  |  |
|  | in or after 2020 | 94.4% | ＜0.001 |  |  |
| Country/region |  |  |  | 0.035 | 0.103 |
|  | Asia | 95.7% | ＜0.001 |  |  |
|  | Europe | 97.0% | ＜0.001 |  |  |
|  | North America | 73.3% | 0.005 |  |  |
|  | South America | / | / |  |  |
| Study outcome |  |  |  | 0.014 | 0.028 |
|  | GERD | 95.0% | ＜0.001 |  |  |
|  | GERS | 95.1% | ＜0.001 |  |  |
| Other obesity  indicators |  |  |  | ＜0.001 | 0.016 |
|  | Yes | 78.8% | ＜0.001 |  |  |
|  | No | 95.7% | ＜0.001 |  |  |
| Sample size |  |  |  | 0.038 | 0.398 |
|  | ≤2000 | 82.0% | ＜0.001 |  |  |
|  | ＞2000 | 98.1% | ＜0.001 |  |  |
| Smoking |  |  |  | 0.035 | 0.103 |
|  | Yes | 96.8% | ＜0.001 |  |  |
|  | No | 82.7% | ＜0.001 |  |  |
|  | None | 95.7% | ＜0.001 |  |  |
| Education level |  |  |  | 0.003 | 0.523 |
|  | Yes | 75.4% | ＜0.001 |  |  |
|  | No | 96.8% | ＜0.001 |  |  |
|  | None | 95.7% | ＜0.001 |  |  |
| Dietary habits |  |  |  | 0.030 | 0.042 |
|  | Yes | 95.2% | ＜0.001 |  |  |
|  | No | 94.9% | ＜0.001 |  |  |
|  | None | 95.7% | ＜0.001 |  |  |
| Medication history |  |  |  | 0.003 | 0.005 |
|  | Yes | 87.2% | ＜0.001 |  |  |
|  | No | 94.2% | ＜0.001 |  |  |
|  | None | 95.7% | ＜0.001 |  |  |
